# Removal of peptidoglycan and inhibition of active cellular processes leads to daptomycin tolerance in *Enterococcus faecalis*

**DOI:** 10.1371/journal.pone.0254796

**Published:** 2021-07-23

**Authors:** Rachel D. Johnston, Brittni M. Woodall, Johnathan Harrison, Shawn R. Campagna, Elizabeth M. Fozo

**Affiliations:** 1 UT-ORNL Graduate School of Genome Science and Technology, University of Tennessee, Knoxville, TN, United States of America; 2 Department of Chemistry, University of Tennessee, Knoxville, TN, United States of America; 3 Department of Microbiology, University of Tennessee, Knoxville, TN, United States of America; 4 Biological and Small Molecule Mass Spectrometry Core, University of Tennessee, Knoxville, TN, United States of America; Universidade Nova de Lisboa, PORTUGAL

## Abstract

Daptomycin is a cyclic lipopeptide antibiotic used in the clinic for treatment of severe enterococcal infections. Recent reports indicate that daptomycin targets active cellular processes, specifically, peptidoglycan biosynthesis. Within, we examined the efficacy of daptomycin against *Enterococcus faecalis* under a range of environmental growth conditions including inhibitors that target active cellular processes. Daptomycin was far less effective against cells in late stationary phase compared to cells in exponential phase, and this was independent of cellular ATP levels. Further, the addition of either the *de novo* protein synthesis inhibitor chloramphenicol or the fatty acid biosynthesis inhibitor cerulenin induced survival against daptomycin far better than controls. Alterations in metabolites associated with peptidoglycan synthesis correlated with protection against daptomycin. This was further supported as removal of peptidoglycan induced physiological daptomycin tolerance, a synergistic relation between daptomycin and fosfomycin, an inhibitor of the fist committed step peptidoglycan synthesis, was observed, as well as an additive effect when daptomycin was combined with ampicillin, which targets crosslinking of peptidoglycan strands. Removal of the peptidoglycan of *Enterococcus faecium*, *Staphylococcus aureus*, and *Bacillus subtilis* also resulted in significant protection against daptomycin in comparison to whole cells with intact cell walls. Based on these observations, we conclude that bacterial growth phase and metabolic activity, as well as the presence/absence of peptidoglycan are major contributors to the efficacy of daptomycin.

## Introduction

As therapeutic strategies have been developed for treating bacterial infections over the past century, there has been a subsequent rise in antibiotic resistant bacteria. Among the CDC’s Resistance Threats in the United States are Gram-positive, vancomycin-resistant enterococci (VRE) [1]. VRE are of particular concern because of the lack of successful treatment strategies available for these pathogens. In the clinic, the Gram-positive targeting lipopeptide daptomycin has been used to treat VRE when other strategies fail. However, due to an abundance of contradictory data, an air of mystery has surrounded the mechanism of action of daptomycin since its discovery in the 1980s. Over the course of its characterization, three main modes of action have been proposed: inhibition of peptidoglycan synthesis, inhibition of lipoteichoic acid (LTA) biosynthesis, and disruption of membrane potential.

Peptidoglycan was proposed as the target of daptomycin, as treatment of both *Staphylococcus aureus* and *Bacillus megaterium* with the antibiotic resulted in a failure to incorporate L-alanine and L-lysine into peptidoglycan in either species [2], and cellular levels of lipid II precursors, needed components for peptidoglycan formation, were increased [3]. Gross cell wall changes were also correlated in some studies with daptomycin resistance and daptomycin treatment in *S*. *aureus* [4,5], *Enterococcus faecalis* [5–8], and *Enterococcus faecium* [8], though this was not true in all cases [9]. However, peptidoglycan was dismissed as a target for daptomycin as removal of the cell wall (generating protoplasts) of *E*. *faecium* did not afford the cells protection against the drug [10]. Recent evidence in *S*. *aureus*, though, supports a model for daptomycin targeting peptidoglycan biosynthesis, as daptomycin was shown to bind to lipid II in the presence of the membrane lipid phosphatidylglycerol, and that other lipid II binding molecules block daptomycin from interacting with lipid II [11].

Older data initially pointed towards LTA as a potential target, as daptomycin was found to inhibit LTA biosynthesis in *S*. *aureus* and *E*. *faecium* [10,12], and LTA precursors within the cell were found to decrease over time following exposure to daptomycin [13]. However, LTA biosynthesis as the target was later abandoned, as daptomycin inhibited not only the production of LTA, but also that of lipids and RNA [14].

Given that daptomycin contains an acyl tail, many speculated that the cell membrane was the true target of the drug. *In vitro* studies using giant unilamellar vesicles demonstrated that daptomycin formed oligomers [15], caused protrusions within vesicles [16], and led to aggregation of lipid bound to daptomycin on the outer surface of the vesicle [16]. Several *in vivo* studies further found that daptomycin interacted with phosphatidylglycerol [17,18], localized to the cell membrane [18], formed pores in the membrane [19], and dissipated membrane potential [19–23]. Importantly, many of the supportive *in vivo* studies for a membrane target of daptomycin were conducted using drug concentrations well above (~7–10x) the minimal inhibitory concentration (MIC) of the bacterium being tested or at late time points [17,20,22,23].

Combining these lines of evidence together, a study in *B*. *subtilis* found that daptomycin associated with fluid regions of the membrane (regions of increased fluidity; RIFs) and rigidified these regions [24]. This rigidification, potentially occurring after binding of daptomycin to lipid II and its precursors [11], occluded peripheral membrane proteins involved in cell wall and phospholipid biosynthesis [24]. The binding of daptomycin to lipid II and its precursors, along with subsequent delocalization of cell wall and lipid synthesis proteins at the membrane would negatively impact these cellular processes, likely contributing to lethality.

Regardless of the specific target of daptomycin, the evidence from the studies summarized above supports that daptomycin targets active cellular processes. Many of the studies of daptomycin demonstrated that treatment with this drug causes biphasic killing of cells [10,22,25–29], a hallmark of persistence [30,31]. As demonstrated by Hobby and Bigger, when bactericidal antibiotics are given at doses above the MIC, they lead to rapid killing of actively growing cells while a genetically identical subset of cells, called persister cells, survive [30,31]. Persister cells can arise through varying mechanisms–including induction of metabolic dormancy through varying expression of the toxin component of toxin/antitoxin systems [32–37] and induction of stress responses [38–44]. In each of these cases, the persister cells in question are not genetically resistant, as they do not grow during treatment with the drug [45]: rather, they are able to survive antibiotic pressure through differential expression of genes which can be stochastic [32,46] or triggered by environmental changes [38–44]. On removal of the antibiotic from the media, these persister cells are then able to grow [47–49].

While some cells may be able to survive treatment with high dosages of drug, survival alone does not indicate true genetic resistance, which results in a higher MIC compared to susceptible cells, along with active growth in the presence of antibiotic [45]. Cells that can survive antibiotic treatment without an increase in the MIC compared to susceptible cells are deemed tolerant [45]. Given that there are subpopulations of cells that survive high daptomycin concentrations [10,22,25–29,50], we examined the role of active cellular processes in daptomycin tolerance through comparison of survival of cells in exponential phase, which are metabolically active and capable of stochastic switching to persister cells [32,46,47] to cells in stationary phase, which have reduced metabolic activity as well as a large population of persisters [51,52]. Comparison of exponential to stationary phase cells is of particular interest, as cells in stationary phase have increased peptidoglycan turnover [53] and thicker peptidoglycan (for *E*. *coli* [54]), a phenomenon also associated with daptomycin resistance and treatment [4–8]. We observed significantly increased daptomycin tolerance as monitored by the number of survivors following transient exposure to high dosage of the drug (7.5-10x MIC) within stationary phase cells. We also tested whether protein and fatty acid biosynthesis were necessary for survival under high concentrations of daptomycin and found that inhibition of these processes significantly increased survival on treatment with daptomycin. Additionally, we observed metabolic changes within stationary phase cells and cells in which protein or fatty acid biosynthesis were inhibited pointed towards altered peptidoglycan metabolism. We removed peptidoglycan from cells and found that these cells were significantly more tolerant to daptomycin treatment. Combined, we demonstrate that inhibition of active growth of *E*. *faecalis* serves to increase its ability to survive high doses of daptomycin, supporting a model for daptomycin targeting peptidoglycan biosynthesis.

## Materials and methods

### Bacterial growth conditions

Bacterial strains used in the study are listed in S1 Table. *E*. *faecalis*, *E*. *faecium*, and *S*. *aureus* strains were grown statically in brain heart infusion (BHI) medium at 37°C unless indicated otherwise. *B*. *subtilis* cultures were grown in LB with shaking (250rpm) at 37°C. Overnight growth cultures were diluted to a final OD_600_ of 0.01 from overnight cultures and harvested at either exponential phase (OD_600_ ~ 0.3) or late stationary phase (24 h after dilution). For examining the effects of *de novo* protein biosynthesis or fatty acid biosynthesis, chloramphenicol (15 μg/mL) or cerulenin (10 μg/mL), respectively, were added to cultures upon reaching OD_600_ ~ 0.225–0.250 (chloramphenicol) or OD_600_ ~ 0.100–0.125 (cerulenin). Cultures were exposed to chloramphenicol for 30 min or cerulenin for 90 min to ensure cellular stasis, with stasis confirmed by determining colony forming units pre- and post-treatment (S1 and S2 Figs).

### Minimal inhibitory concentration (MIC) determination

To perform MIC assays, cells were diluted to a final OD_600_ of 0.01 from overnight cultures into 2 mL of media containing 1.5 mM CaCl_2_ and daptomycin (0, 0.25, 0.5, 1, 2, 4, and 8 μg/mL), and grown for 24 hours prior to determine the minimal inhibitory concentration (S2 Table). Minimum of *n* = 3 for all experiments.

### Cell survival assays

For all daptomycin and SDS challenge assays, cellular survival was determined *via* enumeration of colony forming units. Daptomycin challenge concentrations were chosen to reflect ~7.5-10X the MIC of the respective organisms (S2 Table). Cells were harvested via centrifugation at 2739*xg* for 10 min. Where indicated, the cells were washed with either isotonic buffer (20% sucrose, 0.145M NaCl, 50mM Tris-HCl) [55] or 1x phosphate buffered saline solution (PBS) [25] before resuspension in challenge medium. Daptomycin challenge cultures were set up as follows: for *E*. *faecalis* and *E*. *faecium*, daptomycin was added at 30 μg/mL to cells suspended in BHI with 1.5mM calcium chloride or isotonic buffer containing 1.5mM calcium chloride [50]. For *S*. *aureus* and *B*. *subtilis* cultures, daptomycin was added at 5 μg/mL. Serial dilutions were plated onto BHI agar (*E*. *faecalis*, *E*. *faecium*, and *S*. *aureus*) or LB agar (*B*. *subtilis*) at time zero (immediately prior to daptomycin or SDS addition) and at the indicated time points post-treatment. Colony forming units were enumerated after 18–24h of incubation at 37°C. SDS challenge medium consisted of BHI containing 20% sucrose as an osmoprotective agent and 0.05% SDS. Colony forming units at all time points were normalized to the colony forming units at time zero (before addition of daptomycin or SDS to the culture). Minimum of *n* = 3 for all experiments. Raw CFU data are in S3 Table.

### ATP depletion and tracking

ATP levels were determined as indicated in the text using the BacTiterGlo kit (Promega). For ATP depletion assays, *E*. *faecalis* cells were grown to an OD_600_ of ~0.25. Sodium arsenate dibasic heptahydrate (arsenate; Sigma-Aldrich) was added as indicated in the text to a final concentration of 10 mM, or an equivalent volume of water (solvent control) was added to the cells [56,57]. The cells were incubated for 30 min at 37°C, harvested by centrifugation at 2739*xg*, washed twice with 1x PBS, then resuspended in BHI prior to use for ATP determination. Colony forming units were determined via plating to conclude arsenate treatment did not result in cell death (S3 Fig). Relative light unit values (RLUs) yielded by the BacTiterGlo kit (Promega) were normalized to colony forming units taken at the time the ATP assay was performed. Minimum of *n =* 3 for all experiments.

### Daptomycin synergy

To determine synergy between daptomycin and other antibiotics, a microtiter plate checkerboard assay was performed [58–60]. Overnight cultures were diluted back as above into BHI containing 1.5 mM CaCl_2_ and aliquoted to microplates. Daptomycin (0, 0.25, 0.5, 1, 2, 4, and 8 μg/mL) was added with the concentration increasing along the vertical axis, while the secondary drug was increased along the horizontal axis. The secondary drugs examined were fosfomycin (0, 1, 2, 4, 8, 16, 32, 64, 128, 256, 512 μg/mL), D-cycloserine (0, 2, 4, 8, 16, 32, 64, 128, 256, 512,1024 μg/mL) or ampicillin (0, 0.015625, 0.03125, 0.0625, 0.125, 0.25, 0.5, 1, 2, 4, 8 μg/mL). One row and one column of each plate contained media alone (BHI + 1.5 mM CaCl_2_) to provide a no-growth control. For all experiments, *n =* 3. The fractional inhibitory concentration (FIC) of each drug (drug A and B, respectively) was calculated as follows [60]:

$$FIC_{A}=\frac{MIC_{combined\;drug\;A/B}}{MIC_{drug\;A}}$$ Eq (1)

$$FIC_{B}=\frac{MIC_{combined\;drug\;B/A}}{MIC_{drug\;B}}$$ Eq (2)

The fractional inhibitory concentration index (FICI) was calculated as follows [60]:

$$FICI={FIC}_{A}+{FIC}_{B}$$ Eq (3)

Synergy, additivity, and no interaction are defined as follows: FICI of ≤ 0.5 is defined as synergistic, FICI between 0.5 and 1 is additive, and FICI between 1 and 4 is defined as no interaction [60,61].

### Cell wall removal/protoplast generation

*E*. *faecalis* and *E*. *faecium* were grown in 50 mL BHI to the appropriate growth phase. A whole cell control (4 mL) aliquot and a protoplast (40 mL) aliquot were harvested from the original culture by centrifugation at 2739*xg* for ten min, washed once with isotonic buffer (20% sucrose, 0.145 M NaCl, 50 mM Tris-HCl), then resuspended in 5 mL isotonic buffer [55]. Lysozyme (final concentration of 1 mg/mL) [62] or an equivalent volume of water was added to the appropriate cell mixture. These mixtures were incubated at 37°C for 60 min. Following mock or lysozyme treatment, cells were harvested by centrifugation as above, washed with isotonic buffer, and resuspended in 4 mL isotonic buffer prior to ATP determination (S5 Fig) or were resuspended in 4 mL isotonic buffer containing 1.5 mM CaCl_2_ prior to use for cell survival assays (note that protoplasts were concentrated 10x while mock-treated cells were not). For *S*. *aureus*, cells were treated as above except that lysostaphin was added at 5 μg/mL and DNase was added at 16 μg/mL [55]. *B*. *subtilis* was grown in 100 mL LB to the appropriate growth phase. A whole cell control (4 mL) aliquot and protoplast (80 mL) aliquot were harvested from the original culture by centrifugation as above and resuspended in 4 mL osmoprotective LB (LB supplemented with 20% sucrose, 50 mM Tris-HCl and 0.145 M NaCl). Lysozyme (final concentration of 100 μg/mL) was added to the cells and incubated at 37°C with shaking (250 RPM) for 30 min. Cells were harvested as above, washed with osmoprotective LB and resuspended in an equivalent volume osmoprotective LB containing 1.5 mM CaCl_2_ prior to treatment with daptomycin. Protoplasts and whole cells for each strain were confirmed by Gram-stain.

### Metabolomics

#### Cell harvest and extraction (stationary-phase cells, chloramphenicol- and cerulenin-treated cultures and their respective controls)

Cells were grown as stated above and cell harvest and extraction was adapted from previous literature [63]. Cell culture volumes of 1 mL were harvested by filtration using 0.4 μM membrane filters (Fisher 09-300-71). Dilution plating of the original cell culture was performed at the time of harvest for colony forming unit enumeration. After harvest, the filters were washed with 8 mL of 1x PBS. To begin extraction, filters were immediately placed into respectively labeled petri dishes containing 1.3 mL of prechilled (4°C) extraction solvent (40:40:20 HPLC grade methanol, acetonitrile, water with 0.1% formic acid). Filters were then placed at -20°C for 20 min. Following incubation at -20°C, the filters were washed with 400 μL of extraction solvent, and the solvent was then transferred to respectively labeled 2 mL centrifuge tubes and subjected to centrifugation at 16*xg*. After centrifugation, the supernatant was transferred to a labeled centrifuge tube and the remaining pellet was resuspended in 200 μL of extraction solvent, placed at 4°C for an additional 20 min, subjected to centrifugation as above, and supernatant was added to the above. The samples were then subjected to drying under N_2_. Dried samples were resuspended in 300 μL HPLC water and stored in an autosampler vial at 4°C until analysis.

#### Cell harvest and extraction (whole cells and protoplasts)

As a very low number of protoplasts survived the process of filtration, we elected to harvest cell pellets for whole cell and protoplast cultures (generated as above) prior to extraction. Colony forming units were enumerated at the time of harvest and cell culture volumes of 2 mL for protoplasts (double the volume for whole cells to increase the number of viable cells used for analysis) and 1 mL for whole cells were harvested via centrifugation (15 min 16*xg*) and the supernatant was removed. After harvest, pellets were flash frozen and stored at -80°C prior to extraction. To begin extraction, samples were thawed at 4°C for 30 min. Thawed samples were subjected to the addition of prechilled extraction solvent (0.65 mL) and were shaken at 60 RPM. Afterwards, an additional 0.65 mL of prechilled extraction solvent was added to the samples and the samples were vortexed. Samples containing extraction solvent and cell pellets were chilled for 20 min at -20°C. Samples were then centrifuged for 5 min (16*xg*) and the supernatant was then transferred to new centrifuge tube. An additional 200 μL of extraction solvent was added to cell pellet in the first tube and vortexed. After vortexing the samples were placed at -20°C for an additional 20 min. Samples were centrifuged for an additional 5 min (16*xg*) and the resulting supernatant was removed and placed in the respective tube for drying under N_2_. After drying, samples were resuspended in 300 μL HPLC water and placed into the autosampler at 4°C until analysis.

#### Liquid chromatography mass spectrometry analysis

For liquid chromatography mass spectrometry analysis, 10 μL of sample was injected onto a Synergi 2.5 uM Hydro-RP, 100 Å, 200 x 2.00 mm liquid chromatography column (Phenomenex, Torrance CA) held at 25°C. The data acquisition parameters adapted from Rabinowitz [64] included the operation of the mass spectrometer in full scan mode. The eluent was ionized via electron spray ionization (ESI) in negative mode coupled to a Thermo Scientific Exactive Plus Orbitrap mass spectrometer with the following parameters: spray voltage 3 kV, nitrogen sheath gas 10 (arbitrary units), capillary temperature 320°C, automatic gain control (AGC) target set to 3x10^6 ions. The mass spectrometer was operated at 140,000 resolution with a scan window from 85–800 *m/z* from 0–9 min and 110–1000 *m/z* from 9–25 min. Mobile phase A consisted of 97:3 water methanol 10mM tributylamine, and 15mM acetic acid while solvent B consisted of 100% methanol. Solvent gradient was 0 to 5 min: 0% B, 5–13: min 20% B, 13–15.5 min: 55% B 15.5–19 min 95% B and 19 to 25 min 0% B with a flow rate of 200 μL/min.

#### Data processing

Raw data files generated by Xcalibur were converted to.mzML format using the MS convert from ProteoWizard. El MAVEN was utilized to integrate areas under the curve for known metabolite masses (selected based on our validated standard library of 1080 metabolites) matched with retention time with a mass error of less than 5 ppm. Base background signal was removed, and integrated peak areas were normalized by the ratio of colony forming units of experimental versus control group for each paired control and treatment replicate. Heatmaps were constructed by determining the ratio of normalized, log transformed intensities of experimental values over control values. A paired student’s t-test was utilized to determine significance. PLSDA plots were constructed utilizing Metaboanalyst (metaboanalyst.ca) software. *n* = 5 replicates were performed for all metabolomics experiments with the exception of removal of one replicate from the chloramphenicol vs. ethanol experiment due to technical error, resulting in *n* = 4 replicates in that experiment.

### Statistical analysis

All statistical analyses presented within the text, except where noted, were performed using two-tailed Welch’s *t*-tests, using α = 0.05. For cell survival assays, data were only compared within (not between) individual time points. For each described assay, a minimum of *n* = 3 biological replicates were used. *P-*values for cell survival assays and ATP assays are reported in S4 Table. In figures, *P*-values are represented in ranges as follows: ^ns^ *P* > 0.05; * *P* = 0.05–0.01; ** *P* = 0.01–0.001; *** *P* ≤ 0.001.

## Results

### The sensitivity of *E*. *faecalis* to daptomycin is dependent upon its growth phase

During exponential phase, cells are in a state of active growth and division. However, upon reaching stationary phase, metabolic activity is altered, resulting in physiological changes including thickening of the cell wall [54], reduced protein production [65,66], and often, an increase in tolerance to antibiotics which target active cellular processes [26,33]. To determine whether *E*. *faecalis* OG1RF stationary phase cells have increased tolerance, i.e., increased survival, to daptomycin when compared to exponential phase cells, we examined sensitivity to daptomycin using cell survival assays (via enumeration of colony forming units over time; see Materials and methods). For these experiments, daptomycin concentration was ~7.5–10x MIC (S2 Table).

**Fig 1:**
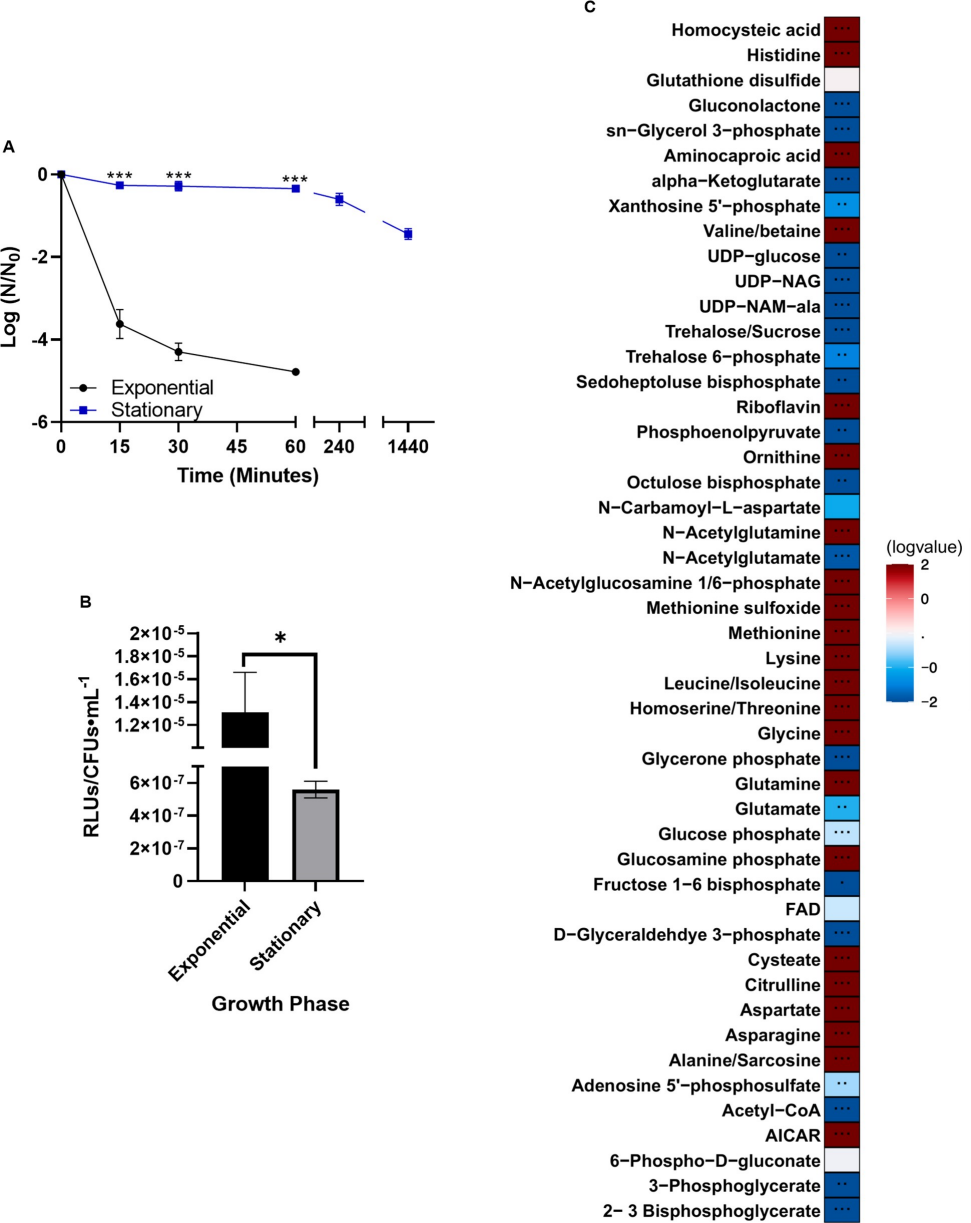
Cells in stationary phase show increased daptomycin tolerance and an altered metabolic profile. (A) Cells were treated with 30 μg/mL daptomycin as described in Materials and Methods. Shown are the average log ratios of colony forming units ± standard deviations for *n* = 3 replicates. *** *P* < 0.001. Note, cell survival for exponential phase cultures was below the limits of detection at 4 hours and beyond. (B) ATP levels of cells were measured using the Promega BacTiterGlo kit. Shown are the average relative light units (RLUs) normalized to colony forming units ± standard deviations for *n* = 3 replicates. * *P* 0.05–0.01; exact *P*-values are found in S4 Table. (C) Metabolites of cells were extracted and detected via mass spectrometry as outlined in Materials and methods. Represented are the log-transformed fold changes of normalized stationary phase cell metabolites to normalized exponential phase cell metabolites. Normalization was performed by dividing raw metabolite data by the ratio of colony forming units of stationary versus exponential phase cells. *n* = 5 replicates.

Stationary phase cultures survived far better when exposed to the drug compared to cells in exponential phase (Fig 1A; *P* < 0.001 after 15 minutes of daptomycin treatment). We noted rapid killing of exponential phase cells within the first 15 min of daptomycin exposure, with a reduction in killing rate over the first hour of treatment. At four hours post-exposure, cells in exponential phase were below the limits of detection, while 3.74% of the cells in stationary phase were still present even after 24 hours of daptomycin exposure (Fig 1A and S3 Table).

Cells in exponential phase generally have higher ATP levels than do their stationary-phase counterparts, likely in order to support the active processes involved in cell division [67]. Further, as reduced ATP levels have been implied in the formation of antibiotic tolerant cells [46,56,68], we measured ATP in both exponential and stationary phase OG1RF cultures. As shown in Fig 1B, stationary phase OG1RF cells had approximately 22-fold less ATP than their exponential phase counterparts when normalized to colony forming units (*P* = 0.024; S4 Table).

As noted, metabolic state is altered when cells are in stationary versus exponential phase [69–72]. To confirm an altered metabolic profile, we performed an untargeted metabolomic approach (Fig 1C). Cells were grown as described above and used for metabolic analyses: note that metabolic peak values were normalized to colony forming units (see Materials and methods) to ensure appropriate comparisons. Of interest, we noted compounds with mass/charge ratios corresponding to peptidoglycan precursors, UDP-NAG (UDP-N-acetylglucosamine) and UDP-NAM-Ala (UDP-N-acetylglucosamine-alanine) were reduced, suggesting that peptidoglycan synthesis was reduced. We also observed alterations in many mass/charge ratios corresponding to amino acids, indicative of altered rates of protein degradation and synthesis [73], unsurprising for cells in stationary phase.

**Fig 2:**
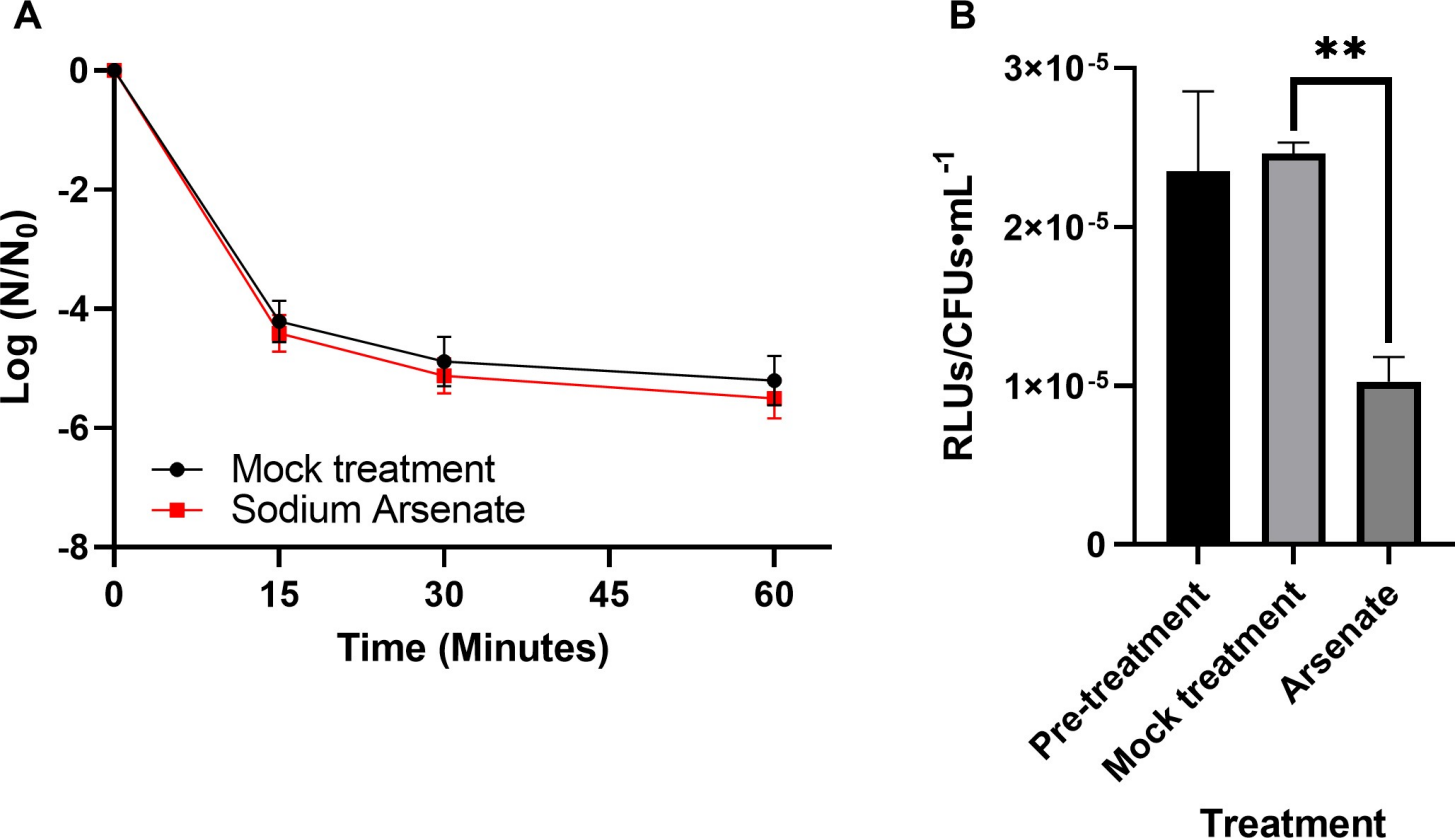
ATP depletion does not induce daptomycin tolerance. (A) Cells grown to exponential phase were treated with 10mM sodium arsenate or mock-treated for 30 min, then subjected to 30 μg/mL daptomycin. Shown are the average log ratios of colony forming units ± standard deviations for *n* = 3 replicates; exact *P*-values are found in S4 Table. (B) ATP levels of pre-treated, mock-treated or arsenate-treated cells (30 min) were measured using the Promega BacTiterGlo kit. Shown are the average relative light units (RLUs) normalized to colony forming units ± standard deviations for *n* = 3 replicates. * *P* ≤ 0.05; exact *P*-values are found in S4 Table.

To determine whether the increased survival of cells in late stationary phase was due to reduced ATP content, ATP depletion [56,57] was performed using sodium arsenate. Importantly, treatment with sodium arsenate did not affect cell survival (S3 Fig and S3 Table), but did reduce ATP levels in exponential phase cells (Fig 2B; *P* = 0.001). Cells treated with arsenate, however, survived daptomycin treatment in a similar fashion to the solvent control (Fig 2A and S3 Table; *P* = 0.769 after 15 minutes of daptomycin exposure). Thus, reduced ATP levels did not correlate with an increase in daptomycin tolerance.

### Inhibition of active cellular processes protects OG1RF from daptomycin

**Fig 3:**
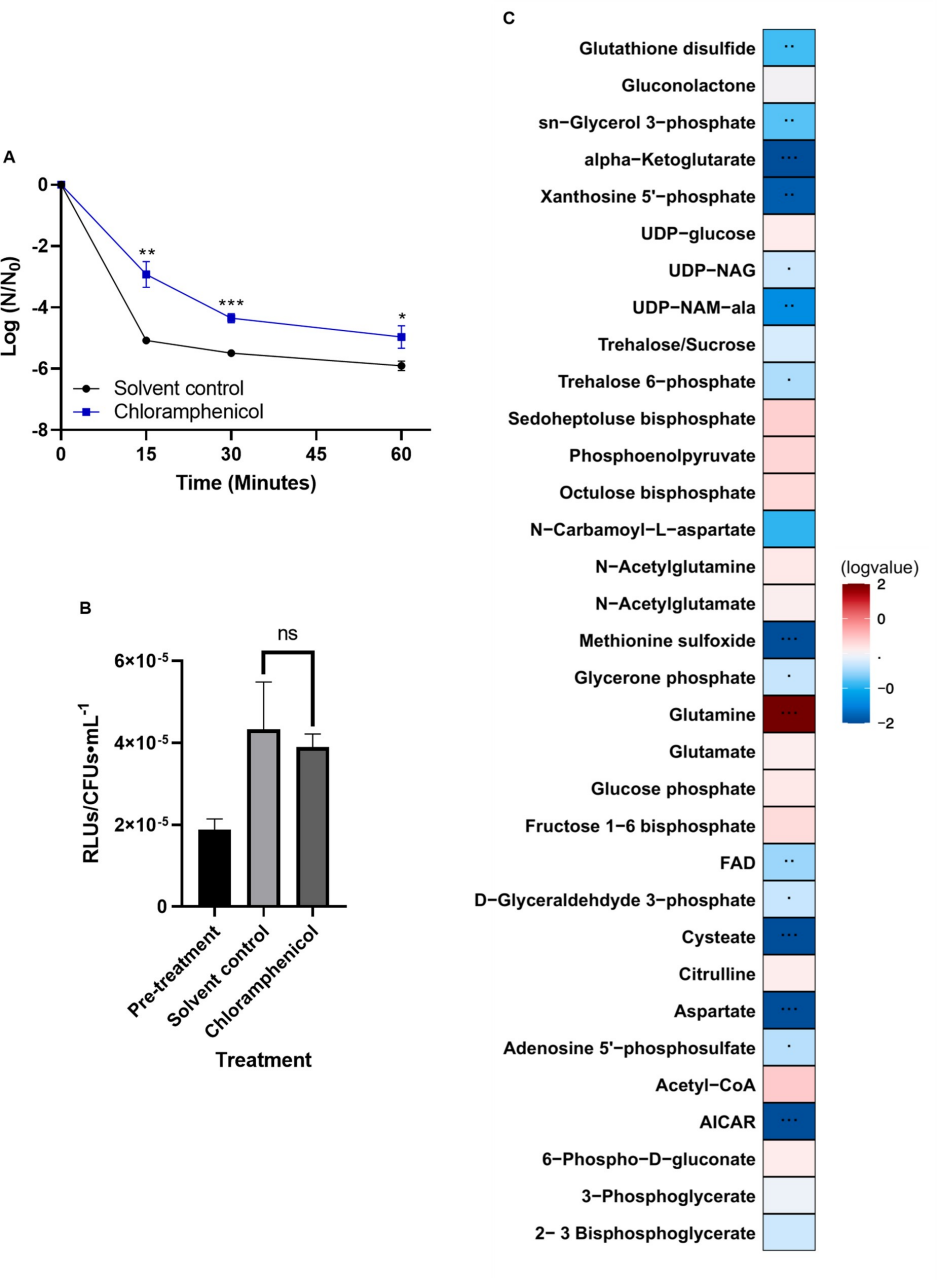
Chloramphenicol-treated cells have increased tolerance to daptomycin and an altered metabolic profile. (A) Cells were grown to an OD_600_ of ~0.250 and treated for 30 min with chloramphenicol or solvent control, washed with 1xPBS, and then exposed to 30 μg/mL daptomycin. Shown are the average log ratios of colony forming units ± standard deviations for *n* = 3 replicates. * *P* = 0.05–0.01; ** *P* = 0.01–0.001; *** *P* ≤ 0.001; NS = no significance; exact *P*-values are found in S4 Table. (B) ATP levels of pre-treated, solvent-treated or chloramphenicol-treated cells were measured using the Promega BacTiterGlo kit. Shown are the average relative light units (RLUs) normalized to colony forming units ± standard deviations for *n* = 3 replicates. ns = *P* > 0.05; exact *P*-values are found in S4 Table (C) Metabolites of cells were extracted and detected via mass spectrometry as outlined in Materials and methods. Represented are the log-transformed fold changes of normalized chloramphenicol-treated cell metabolites to normalized solvent control-treated cell metabolites. Normalization was performed by dividing raw metabolite data by the ratio of colony forming units of chloramphenicol-treated versus solvent control-treated cells. *n* = 4 replicates.

Survival of a tolerant population has been attributed to a variety of mechanisms, including a reduction in cellular growth [47]. As stationary phase OG1RF cells survived daptomycin treatment far better than exponential cells, we hypothesized that reduced cellular growth could induce daptomycin tolerance. We artificially induced growth stasis through inhibition of either *de novo* protein biosynthesis via addition of chloramphenicol or *de novo* fatty acid biosynthesis via addition of cerulenin. Inhibition of cellular growth was used as a proxy for inhibition of either process (S1 and S2 Figs). Upon addition of chloramphenicol, cellular growth, as observed by colony forming units, was halted (S1 Fig) but ATP levels were comparable to solvent control-treated cultures (Fig 3B; *P =* 0.59). Yet, chloramphenicol treatment induced protection from daptomycin, particularly during the first 15 min of treatment (Fig 3A and S3 Table; 1.8 log fold change; *P* = 0.002 after 15 minutes of daptomycin exposure). This protective effect of pre-treatment with chloramphenicol was not as great as the protection observed in cells treated with daptomycin during late stationary phase, which, when compared with their exponential phase counterparts, had a 4.1 log fold increase in survivors during the first 15 min of treatment (Fig 1A).

We hypothesized that protection induced by chloramphenicol may be due to alteration in the metabolic profile upon the inhibition of cellular growth. Using metabolomics, we detected numerous species with altered ratios when examining chloramphenicol versus mock-treated cultures (normalized to colony forming units, please see Materials and methods, Fig 3C). In the chloramphenicol-treated cultures, we did note statistically significant decreases in mass/charge ratios corresponding to UDP-NAG and UDP-NAM-Ala, a cytoplasmic precursor to the membrane-bound peptidoglycan precursors lipid I and lipid II, relative to solvent control-treated cultures (Fig 3C), along with statistically significant alterations to mass/charge ratios corresponding to different amino acids, including glutamine, unsurprising given that chloramphenicol inhibits protein synthesis.

**Fig 4:**
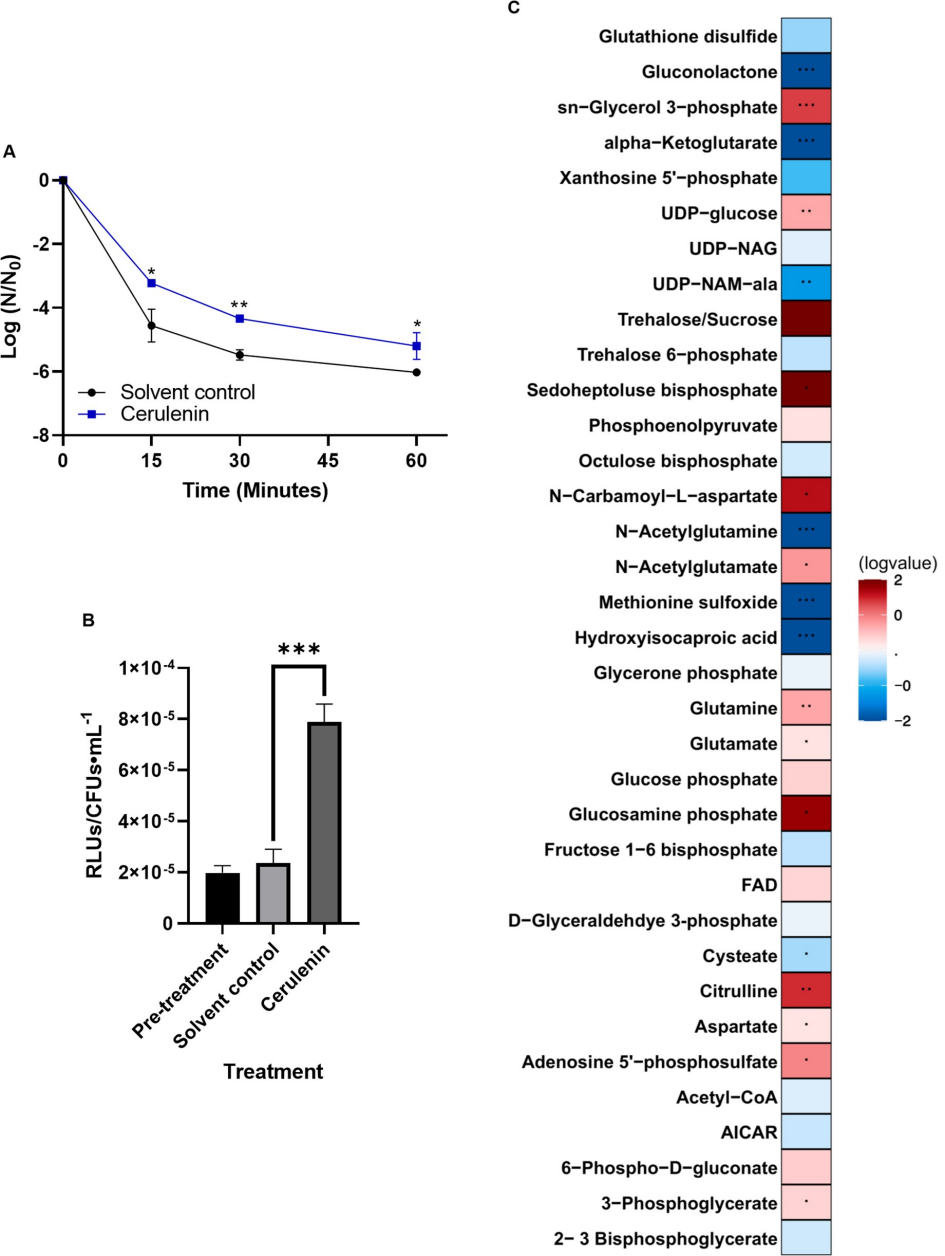
Cerulenin-treated cells have increased tolerance to daptomycin and an altered metabolic profile. (A) Cells were grown to early exponential phase, treated with cerulenin or solvent control, washed with 1x PBS, and then subjected to treatment with 30 μg/mL daptomycin. Shown are the average log ratios of survivors ± standard deviations for *n* = 3 replicates. * *P* = 0.05–0.01; ** *P* = 0.01–0.001; exact *P*-values are found in S4 Table. (B) ATP levels of pre-treated, solvent-treated and cerulenin-treated cells were measured using the Promega BacTiterGlo kit. Shown are the average relative luminescence units (RLUs) normalized to colony forming units ± standard deviations for *n* = 3 replicates. *** *P* ≤ 0.001; exact *P*-values are found in S4 Table. (C) Metabolites of cells were extracted and detected via mass spectrometry as outlined in Materials and methods. Represented are the log-transformed fold changes of normalized cerulenin-treated cell metabolites to normalized solvent control-treated cell metabolites. Normalization was performed by dividing raw metabolite data by the ratio of colony forming units of cerulenin-treated versus solvent control-treated cells. *n* = 5 replicates.

We also examined the effects of cellular growth stasis on daptomycin sensitivity by inhibiting membrane fatty acid biosynthesis via cerulenin treatment. Upon treatment of cells with cerulenin, cell growth was halted as observed via colony forming units (S2 Fig) and there was a 1.1 log fold protection induced relative to the solvent control (Fig 4A and S3 Table; *P* = 0.022 after 15 minutes of daptomycin treatment). However, unlike treatment with chloramphenicol, treatment with cerulenin resulted in increased ATP levels (Fig 4B; *P* < 0.001).

Given these findings, we examined the metabolic profile of cerulenin-treated cells versus their solvent controls, in order to identify potential metabolic signatures conserved between chloramphenicol and cerulenin treatment (Fig 4C). We again observed a decrease in the mass/charge ratio corresponding to UDP-NAM-Ala, but in this case, an insignificant decrease in detected UDP-NAG. Unique to cerulenin-treated cultures, we also observed a buildup of the mass/charge ratio corresponding to *sn*-Glycerol-3-phosphate (G3P), the precursor to membrane phospholipids–this is likely because the cerulenin-treated cultures were unable to synthesize fatty acids which are placed on G3P to form lysophosphatidic acid [74].

Interestingly, for all of our experimental treatments (stationary phase, chloramphenicol-treated, and cerulenin-treated cells), we noted decreases in the mass/charge ratio corresponding to UDP-NAM-Ala and alpha-ketoglutarate, a key player in the citrate cycle and a source of glutamine and glutamate, along with an increase in the amino acid glutamine. Importantly, glutamine is not only used in protein synthesis but is also converted to D-glutamate for building peptidoglycan. These changes in these metabolites indicate a dysregulation of cell wall synthesis which we investigated further below.

### Removal of peptidoglycan protects OG1RF from daptomycin

Despite evidence from multiple studies implicating the cell wall, specifically peptidoglycan, as the major target of daptomycin [2,3], additional data supported that membrane targeting and subsequent pore formation were critical for activity of the drug [17–20,22,23]. Given these discrepancies and our own above findings, in particular the significant decrease in the mass/charge ratio corresponding to UDP-NAM-Ala in stationary phase and chloramphenicol- and cerulenin-treated cells, we examined the role of peptidoglycan in the daptomycin sensitivity of OG1RF. We divided exponential cultures such that a portion was mock-treated (remained as whole cells) and the rest were treated to remove peptidoglycan (protoplasts; see Materials and methods).

**Fig 5:**
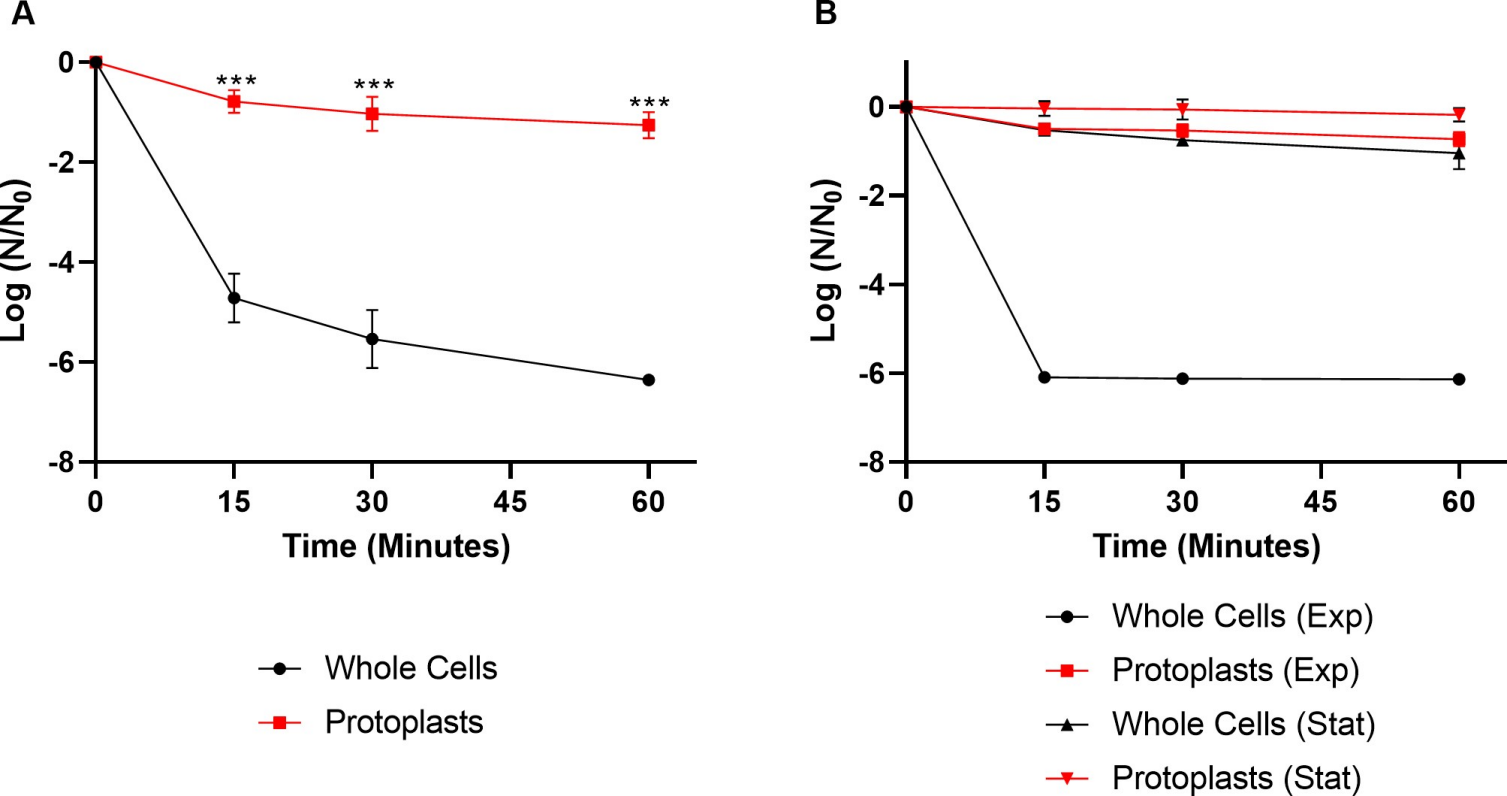
Removal of peptidoglycan increases tolerance to daptomycin in *E*. *faecalis*. (A) Cells were grown to mid-logarithmic phase, split, and treated with either lysozyme to generate protoplasts or solvent control (whole cells) and then treated with 30 μg/mL daptomycin. Shown are the average log ratios of colony forming units ± standard deviations for *n* = 3 replicates. *** *P* ≤ 0.001; exact *P*-values are found in S4 Table. (B) Cells were grown to mid-logarithmic phase (Exp) or to late stationary phase (24 hour cultures; Stat) and treated with either lysozyme to generate protoplasts or solvent control (whole cells) and then treated with 30 μg/mL daptomycin. Shown are the average log ratios of colony forming units ± standard deviations for *n* = 3 replicates.

Upon treatment with daptomycin, when compared with whole-cell counterparts, we note that the protoplasts had at least 1.8 log-fold more colony forming units at every time point (Fig 5A and S3 Table; *P* < 0.001 after 15 minutes of exposure to daptomycin). As removal of peptidoglycan could stimulate a variety of responses, including possible stress-response regulons, we examined survival when cells were treated with SDS, an anionic detergent that disrupts membranes and acts as a non-specific stressor which activates efflux pumps and *rpoS* stress responses [75]. However, removal of peptidoglycan did not induce any protection from SDS (S4 Fig; *P* = 0.222 after 15 minutes of daptomycin treatment) or significant differences in ATP levels (S5 Fig). We also performed metabolomics analyses on protoplast cells compared to whole cells (S6 Fig), and observed increases in many metabolites, including UDP-NAG, however, the variation between biological replicates was very high, resulting in insignificant results for most detected metabolites.

### Daptomycin activity is enhanced with specific peptidoglycan synthesis targeting antibiotics

Our above data suggests that the presence of peptidoglycan renders OG1RF sensitive to daptomycin. As there are a variety of different antibiotics that target unique, specific enzymatic processes in the synthesis of peptidoglycan, we performed a checkerboard assay to better conclude what daptomycin may specifically target. A checkerboard assay measures the effectiveness of two separate antibiotics when combined versus when given independently to an organism to quantitatively conclude whether the drugs are additive or synergistic [58–60]. We examined D-cycloserine, which blocks the interconversion of L-alanine and D-alanine, as D-alanine is found within the peptide chains of peptidoglycan; fosfomycin, which targets the first committed step of synthesis, namely condensation of enolpyruvate with UDP-N-acetylglucosamine by the enzyme MurA; and ampicillin, which inhibits the transpeptidation reaction that crosslinks two peptidoglycan molecules.

We noted that there was no interaction between D-cycloserine and daptomycin, potentially due to the cellular growth conditions (Table 1, see discussion). Fosfomycin, in agreement with previous studies in *E*. *faecium* and *S*. *aureus* [76–79], was synergistic with daptomycin, suggesting that daptomycin impacts early peptidoglycan synthesis steps in *E*. *faecalis* (see discussion). Additionally, we observed that ampicillin was additive when combined with daptomycin (Table 1).

**Table 1:** Combination of peptidoglycan synthesis-targeting antibiotics with daptomycin on *E*. *faecalis* OG1RF.

Combination (antibiotic A–antibiotic B)	Antibiotic A MIC (μg/mL)	Antibiotic B MIC (μg/mL)	Antibiotic B MIC (μg/mL) at Dap MIC of 0.5 μg/mL	FICI	Effect
Dap–FOF	4	32–128	8–32	0.25–0.375	Synergistic
Dap–DCS	2	128	128	1.25	No interaction
Dap–AMP	4	0.5	0.25	.625	Additive

Represented are the ranges of individual antibiotic MICs along with the range of the MICs of antibiotic B combined with daptomycin at 0.5 μg/mL. *n* = 3 for each experiment. FICI ≤ 0.5: Synergistic, FICI = 0.5–1: Additive; FICI = 1–4: No interaction. DAP; daptomycin, FOF; Fosfomycin, DCS; D-cycloserine, AMP; ampicillin.

### Peptidoglycan removal enhances stationary phase cell survival against daptomycin

Combining our above data, we found that stationary phase cells were far more tolerant to daptomycin when compared to their exponential-phase cell counterparts (Figs 1A and 5B), and that removal of peptidoglycan also results in high tolerance (Fig 5 and S3 Table). Given that peptidoglycan turnover is increased in stationary phase relative to exponential phase in *E*. *faecalis* [53], and that in stationary phase *E*. *coli*, peptidoglycan is thicker and comprises a higher percentage of the total dry weight compared to exponential phase cells [54], we decided to probe whether removal of the cell wall of stationary phase cells altered their daptomycin tolerance. We found that there was a small, but significant difference between survival of stationary phase whole cells versus stationary phase protoplasts (Fig 5B and S3 Table; *P* = 0.023 at all time points).

### Removal of peptidoglycan protects multiple Gram-positive bacteria from killing by daptomycin

**Fig 6:**
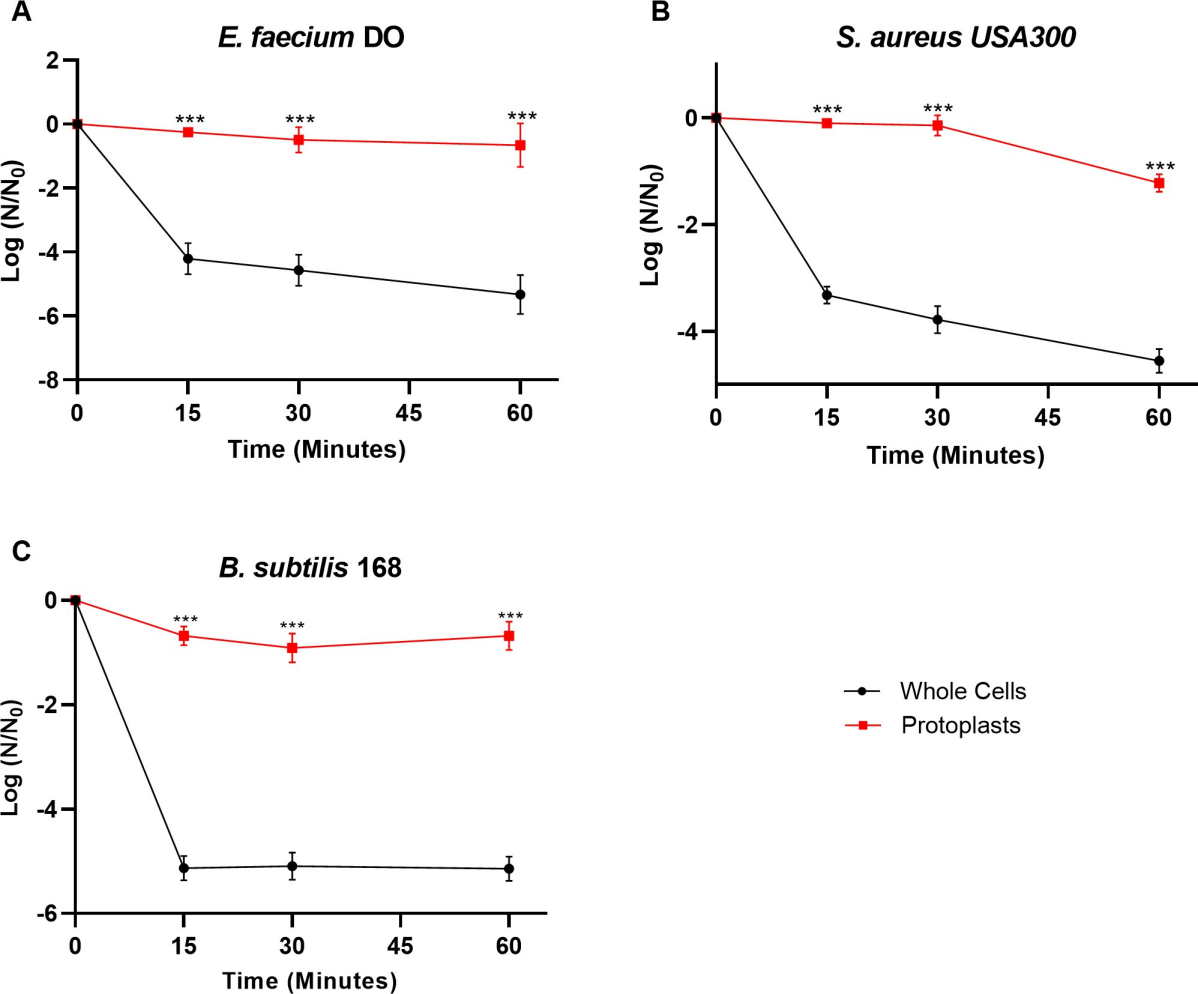
Removal of peptidoglycan increases tolerance to daptomycin in *E*. *faecium*, *S*. *aureus*, and *B*. *subtilis*. (A) Treatment of either *E*. *faecium* whole cells or protoplasts (see Materials and Methods) with 30 μg/mL daptomycin. Shown are the average log ratios of survivors ± standard deviations for *n* = 3 replicates. *** *P* ≤ 0.001; exact *P*-values are found in S4 Table. (B) *S*. *aureus* whole cells or protoplasts (see Materials and Methods) treated with 5 μ/mL daptomycin. Shown are the average log ratios of survivors ± standard deviations for *n* = 3 replicates. *** *P* ≤ 0.001; exact *P*-values are found in S4 Table. (C) *B*. *subtilis* whole cells or protoplasts (see Materials or Methods) treated with 5 μ/mL daptomycin. Shown are the average log ratios of survivors ± standard deviations for *n* = 3 replicates. *** *P* ≤ 0.001; exact *P*-values are found in S4 Table.

As removal of peptidoglycan in *E*. *faecalis* altered daptomycin tolerance, we examined whether its removal in other Gram-positive species results in increased daptomycin survival. Thus, we compared survival of whole cells and protoplasts of *E*. *faecium*, *S*. *aureus*, and *B*. *subtilis* upon treatment with 7.5–10x MIC (S2 Table). We found that protoplasts of *E*. *faecium* had a 2.1 log-fold survival (or more) over whole cell counterparts (Fig 6A; *P* < 0.001 at all time points). Similarly, removal of the peptidoglycan of *S*. *aureus* protoplasts had a minimum 3.4 log-fold increase in survivors (Fig 6B; *P* < 0.001 at all time points) while *B*. *subtilis* protoplasts had a 2.5 log fold increase (or more) in survivors compared to whole cell controls (Fig 6C; *P* < 0.001 at all time points). This demonstrates that across these bacterial species, removal of peptidoglycan negates most killing by daptomycin.

## Discussion

While in the literature there have been multiple proposed targets for daptomycin, several lines of evidence support peptidoglycan biosynthesis being its major target [2–8,11]. Here we demonstrate that daptomycin tolerance, *i*.*e*., increased survival following treatment above the MIC, is dependent on several factors: growth phase, the ability of cells to synthesize proteins and fatty acids, and the presence/absence of peptidoglycan. Combined, these data support that peptidoglycan, which is intricately linked to membrane biosynthesis and requires protein biosynthesis, is necessary for full daptomycin efficacy.

*E*. *faecalis* stationary-phase cells were protected to a high degree against daptomycin relative to their exponential-phase counterparts (Fig 1A). However, similar levels of protection were induced by peptidoglycan removal in exponential phase compared to stationary phase whole cells (Figs 1B and 5B, S3 Table). This is possibly due to cell wall changes which occur in bacteria during stationary phase, including increases in wall thickness [54] and increases in peptidoglycan recycling [80] as well as decreased peptidoglycan synthesis [81]. Additionally, this could be due to a reduction in wall turnover in stationary phase cells compared to exponential phase cells [82] or due to increased degradation of MurA, which catalyzes the first committed step of peptidoglycan synthesis by transferring enolpyruvate from phosphoenolpyruvate to UDP-NAG [83], in stationary phase cells [84]. In our data, we noted that in stationary phase cells, phosphoenolpyruvate was decreased relative to exponential phase cells (Fig 1C), potentially indicating altered MurA activity.

Given these data and previous studies, why would addition of chloramphenicol, a *de novo* protein synthesis inhibitor, or cerulenin, an inhibitor of fatty acid biosynthesis, induce daptomycin protection? The production of peptidoglycan is needed for cellular replication and expansion of the population size. Inhibiting protein biosynthesis, as shown via a halt in colony forming units (S1 Fig), prevents cellular division. However, in *E*. *faecalis* (formerly *Streptococcus faecalis*), previous work indicated that upon treatment with chloramphenicol, cellular division stopped while peptidoglycan synthesis still occurred, though at a lower rate, resulting in thickening of the cell wall [85]. Thus, perhaps the effectiveness of daptomycin may be altered because of a reduction in peptidoglycan synthesis. Similarly, previous studies have shown that addition of the fatty acid biosynthesis inhibitor cerulenin to exponentially growing *E*. *faecalis* reduces but does not abrogate peptidoglycan synthesis [86], and can lead to cell expansion and altered/misplaced cell wall biosynthesis [87]. The protective effect we observe on treating exponentially growing cultures with chloramphenicol or cerulenin could possibly also be explained by a dilution of targets caused by increased and misplaced wall synthesis [85–87].

Our metabolomics data also suggests that decreased peptidoglycan biosynthesis could be the cause of the protective effects we see, as the mass/charge ratio corresponding to UDP-NAM-Ala is decreased in stationary phase cells, cells treated with cerulenin, and cells treated with chloramphenicol (Figs 1C, 3C and 4C). As noted, we also observed a significant decrease in the mass/charge ratio corresponding to alpha-ketoglutarate in stationary phase cells and upon treatment with chloramphenicol and cerulenin, as well as a significant increase in the mass/charge ratio corresponding to glutamine under these conditions. Fascinatingly, both of these metabolites can be linked back to peptidoglycan synthesis, as alpha-ketoglutarate and glutamine are suppliers of glutamate, which is an amino acid incorporated into the amino acid side-chains of peptidoglycan. It should be noted here that in order to confirm these significant metabolites among the experimental groups, a significant analysis microarray (SAM) was performed to calculate potential rates of false discovery and identify those metabolites that are statistically significant based on two different analyses (heatmap and SAM).

The results of these experiments also provide some insights as to why treatment with high levels of daptomycin result in “biphasic” killing of multiple bacterial species [10,22,25–29]. Those cells that survive high daptomycin do so likely because they are not actively synthesizing and turning over peptidoglycan [11,24].

For both chloramphenicol-treated and cerulenin-treated cultures, we also noted a significant decrease in the mass/charge ratio corresponding to methionine sulfoxide, a precursor to methionine. However, due to the lack of corresponding increases of mass/charge ratios of methionine sulfoxide in stationary phase cells, we cannot confirm that this decrease is directly responsible for the increases in daptomycin tolerance we observe under these conditions.

Further evidence to support peptidoglycan as a target for daptomycin comes from the observed positive effects of combining daptomycin with other cell-wall targeting drugs (Table 1). The synergistic effects between fosfomycin and daptomycin could potentially be explained by an upregulation of MurA caused by exposure to daptomycin [88,89]. In turn, fosfomycin tightly binds to MurA, thus blocking the first committed step of peptidoglycan biosynthesis [83]. Similarly, daptomycin was found in *S*. *aureus* to increase the expression of PBP1 [90], the target of ampicillin, thus potentially explaining the observed additive effect between daptomycin and ampicillin which is in line with synergy observed during time-kill experiments combining β-lactam antibiotics with daptomycin [91]. We did not observe an interaction between daptomycin and D-cycloserine: this could be because D-cycloserine simply “freezes” the pools of L-ala and D-ala by binding to alanine racemase. Note that its effect on peptidoglycan synthesis depends on the current state of the pools of L-ala and D-ala [92] and given that our experiments were carried out in rich media, it is possible that the pool of D-ala was great enough to prevent either a synergistic or additive effect between daptomycin and D-cycloserine.

Interestingly, a recent study gave evidence that pre-incubation of *B*. *subtilis* with teixobactin, a lipid II and lipid III targeting antibiotic [78], protected cells against daptomycin, rather than acting synergistically with it [11]. As teixobactin shares a target with daptomycin, a pre-treatment with (and subsequent washing away of, as performed by Grein et al) teixobactin could reduce the number of targets for daptomycin to effectively bind to, leading to protection against daptomycin [11].

While the data presented above support peptidoglycan is a target for daptomycin, it was originally dismissed, as daptomycin still killed *E*. *faecium* cells which had their cell walls removed [10]. Though our observations agree with published literature that protoplasted cells can be subject to killing by daptomycin [10], our inclusion of a whole cell control provides a fuller picture. We found that some killing of protoplasted cells occurred, however, when protoplasts were compared to the whole cell control, there was a minimum of a 1.8 log-fold increase in survival against daptomycin, independent of the bacterial species being tested (Figs 5 and 6).

While we have demonstrated here that bacterial growth state and that the presence or absence of peptidoglycan are of major importance for daptomycin efficacy regardless of bacterial species, it is possible and indeed probable that treatment of cells with lysozyme and with different antibiotics may alter the cellular membrane, in particular phosphatidylglycerol amounts and localization, reducing daptomycin binding. This is critical as daptomycin has been shown to interact with lipid II and phosphatidylglycerol [11,17,18]. Further investigation is needed to determine the bacterial membrane composition under these conditions in order to assess whether specific changes to the bacterial membrane are responsible for altered daptomycin efficacy.

Within this work, we have demonstrated that intact peptidoglycan is required for full daptomycin efficacy, and that dysregulation of cellular metabolism can result in significant increases in daptomycin tolerance. Our results aid in informing of possible effective treatment combinations in the clinic and new therapeutic approaches.

## Supporting information

S1 Fig Addition of chloramphenicol halts cellular division. (A) Optical density of cells over time. Shown are the average optical densities at 600 nm ± standard deviations for *n* = 3 replicates. Black arrow indicates addition of chloramphenicol or solvent control at mid-logarithmic phase (at 175 min into the growth curve; OD_600_ ~ 0.250). Red arrow indicates when cells were harvested and processed prior to a subsequent cell survival assay (30 min post-chloramphenicol/solvent control treatment). (B) The number of colony-forming units as determined by dilution plating of the cultures in S1A. T0 is just prior to the addition of chloramphenicol or solvent control into the cultures, and T30 and T60 are 30 and 60 min after addition of chloramphenicol or solvent control into the cultures, respectively. *n = 3*.
(TIF)

S2 Fig Addition of cerulenin halts cellular division. (A) Optical density of cells over time. Shown are the average optical densities at 600 nm ± standard deviations for *n* = 3 replicates. Black arrow indicates addition of cerulenin or solvent control at early-logarithmic phase (at 125 min into the growth curve; OD_600_ ~ 0.125). Red arrow indicates when cells were harvested and processed prior to a subsequent cell survival assay (90 min post-cerulenin/solvent control treatment). (B) The number of colony-forming units as determined by dilution plating of the cultures in S2A. T0 is just prior to the addition of cerulenin or solvent control into the cultures. T30, T60, T90, and T120 are 30, 60, 90, and 120 min after addition of cerulenin or solvent control into the cultures, respectively. *n = 3*.
(TIF)

S3 Fig Cells treated with sodium arsenate are still viable. *E*. *faecalis* OG1RF cells were grown in BHI until OD_600_ reached ~0.250. The cultures were split and treated with 10mM arsenate or mock-treated with water for 30 minutes. Following treatment, the cells were washed twice to remove arsenate and resuspended in BHI prior to enumerating colony forming units. Represented are the log ratio of colony forming units ±standard deviations over time. ns = not significant. *n* = 3.
(TIF)

S4 Fig Protoplasts are not protected against SDS. *E*. *faecalis* OG1RF cells were grown to mid-logarithmic phase (OD_600_ ~ 0.3–0.4), split and treated with either lysozyme to generate protoplasts or solvent control to leave cell whole. Subsequently washed cells were subjected to killing by 0.05% SDS. Shown are the average log ratios of survivors ± standard deviations for *n* = 3 replicates. No significant differences were observed. Exact *P*-values are reported in S4 Table.
(TIF)

S5 Fig ATP levels of *E*. *faecalis* OG1RF whole cells and protoplasts normalized to colony forming units. Cells were grown to mid-logarithmic phase (OD_600_ ~ 0.3–0.4) and treated with either lysozyme to generate protoplasts or solvent control to leave cells whole. ATP levels of pre-treatment cells and whole cells or protoplasts were measured using the Promega BacTiterGlo kit and normalized to colony forming units. Shown are the average relative light units (RLUs) normalized to colony forming units ± standard deviations for *n* = 3 replicates. ** *P* = 0.01–0.001; exact *P*-values are reported in S4 Table.
(TIF)

S6 Fig Removal of peptidoglycan results in buildup of several metabolites. Exponentially growing (OD_600_ ~ 0.3) *E*. *faecalis* cells were grown to mid-logarithmic phase (OD_600_ ~ 0.3–0.4), split and treated with either lysozyme to generate protoplasts or solvent control to leave cell whole as described in Materials and methods. Metabolites of the cells were extracted and detected via mass spectrometry as outlined in Materials and methods. Represented are the log-transformed fold changes of normalized whole cell metabolites to normalized protoplast metabolites. Normalization was performed dividing raw metabolite data by the ratio of colony forming units of protoplast versus whole cells. *n* = 5 replicates.
(TIF)

S1 Table List of bacterial strains used in this study. (DOCX)

S2 Table Minimal inhibitory concentrations of daptomycin for the bacterium used in this study. Minimal inhibitory concentrations (MICs) were performed using broth dilution technique. The ranges observed are reported above. Brain heart infusion + 1.5 mM CaCl_2_ was used for *Enterococcus* and *Staphylococcus* strains grown statically. LB + 1.5 mM CaCl_2_ was used for *B*. *subtilis* grown shaking (250 RPM). *n* = 3 for all experiments.
(DOCX)

S3 Table Colony forming units per milliliter (CFU/mL) taken during cell survival assays within this study. (DOCX)

S4 Table *P-*values determined within this study. Two-tailed Welch’s *t-*tests were used to generate *P*-values. In the text, α = 0.05 was used to determine significance.
(DOCX)
