# Supplementary figures and images for: Removal of peptidoglycan and inhibition of active cellular processes leads to daptomycin tolerance in Enterococcus faecalis
Source: PLoS One. 2021 Jul 23;16(7):e0254796. doi: 10.1371/journal.pone.0254796 (PMC8301656; doi:10.1371/journal.pone.0254796)

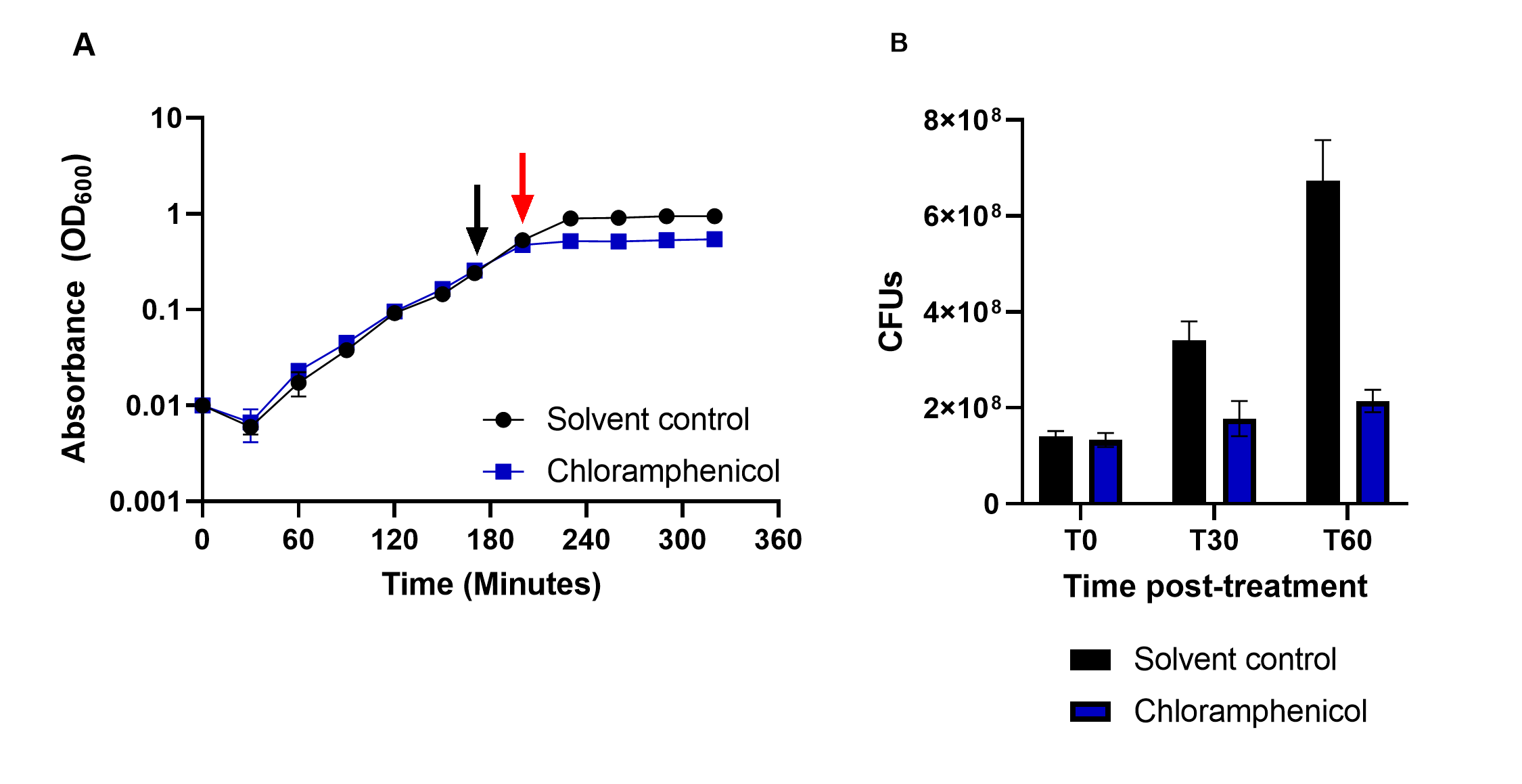

Supplement: S1 Fig — (A) Optical density of cells over time. Shown are the average optical densities at 600 nm ± standard deviations for n = 3 replicates. Black arrow indicates addition of chloramphenicol or solvent control at mid-logarithmic phase (at 175 min into the growth curve; OD600 ~ 0.250). Red arrow indicates when cells were harvested and processed prior to a subsequent cell survival assay (30 min post-chloramphenicol/solvent control treatment). (B) The number of colony-forming units as determined by dilution plating of the cultures in S1A. T0 is just prior to the addition of chloramphenicol or solvent control into the cultures, and T30 and T60 are 30 and 60 min after addition of chloramphenicol or solvent control into the cultures, respectively. n = 3. (TIF) [file pone.0254796.s001.tif]

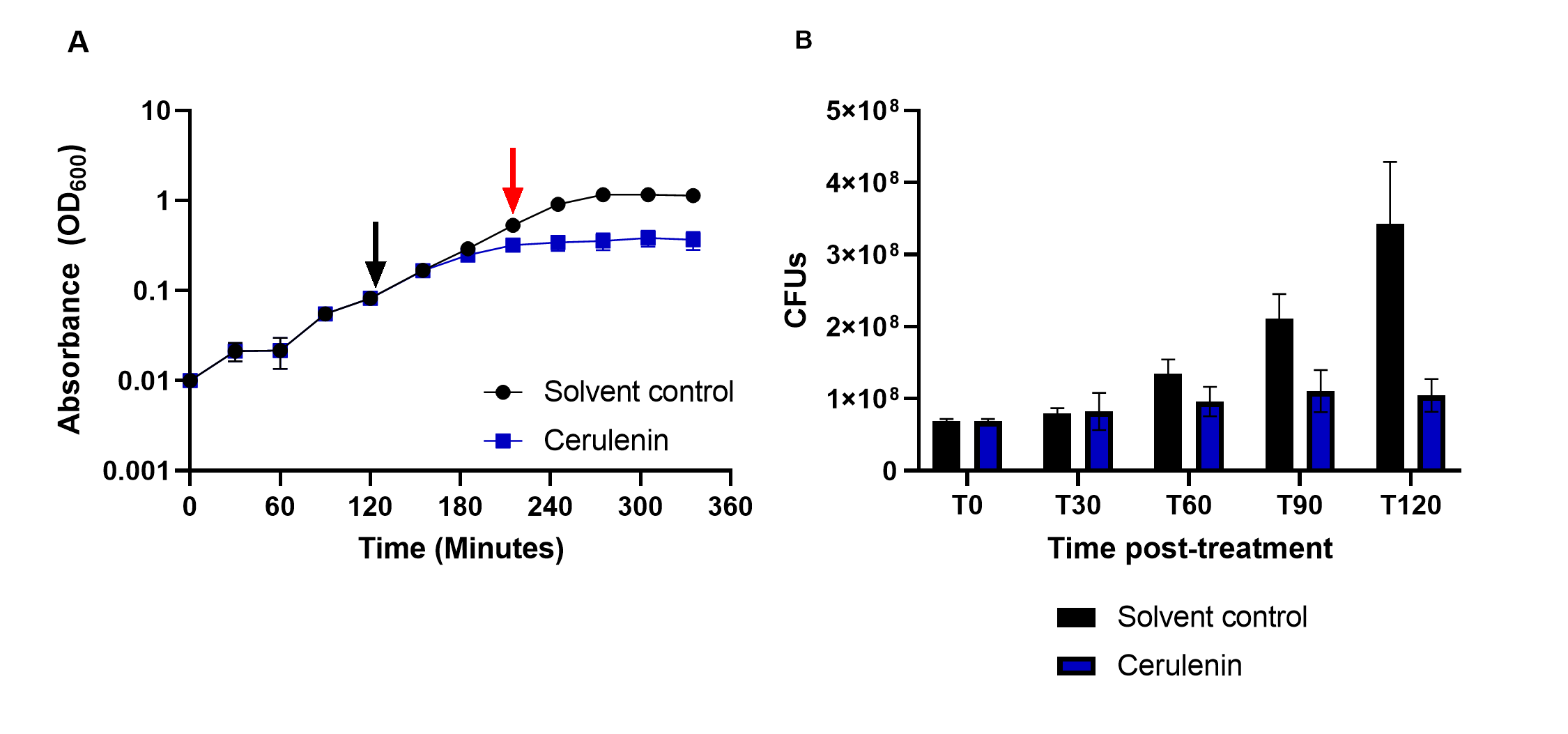

Supplement: S2 Fig — (A) Optical density of cells over time. Shown are the average optical densities at 600 nm ± standard deviations for n = 3 replicates. Black arrow indicates addition of cerulenin or solvent control at early-logarithmic phase (at 125 min into the growth curve; OD600 ~ 0.125). Red arrow indicates when cells were harvested and processed prior to a subsequent cell survival assay (90 min post-cerulenin/solvent control treatment). (B) The number of colony-forming units as determined by dilution plating of the cultures in S2A. T0 is just prior to the addition of cerulenin or solvent control into the cultures. T30, T60, T90, and T120 are 30, 60, 90, and 120 min after addition of cerulenin or solvent control into the cultures, respectively. n = 3. (TIF) [file pone.0254796.s002.tif]

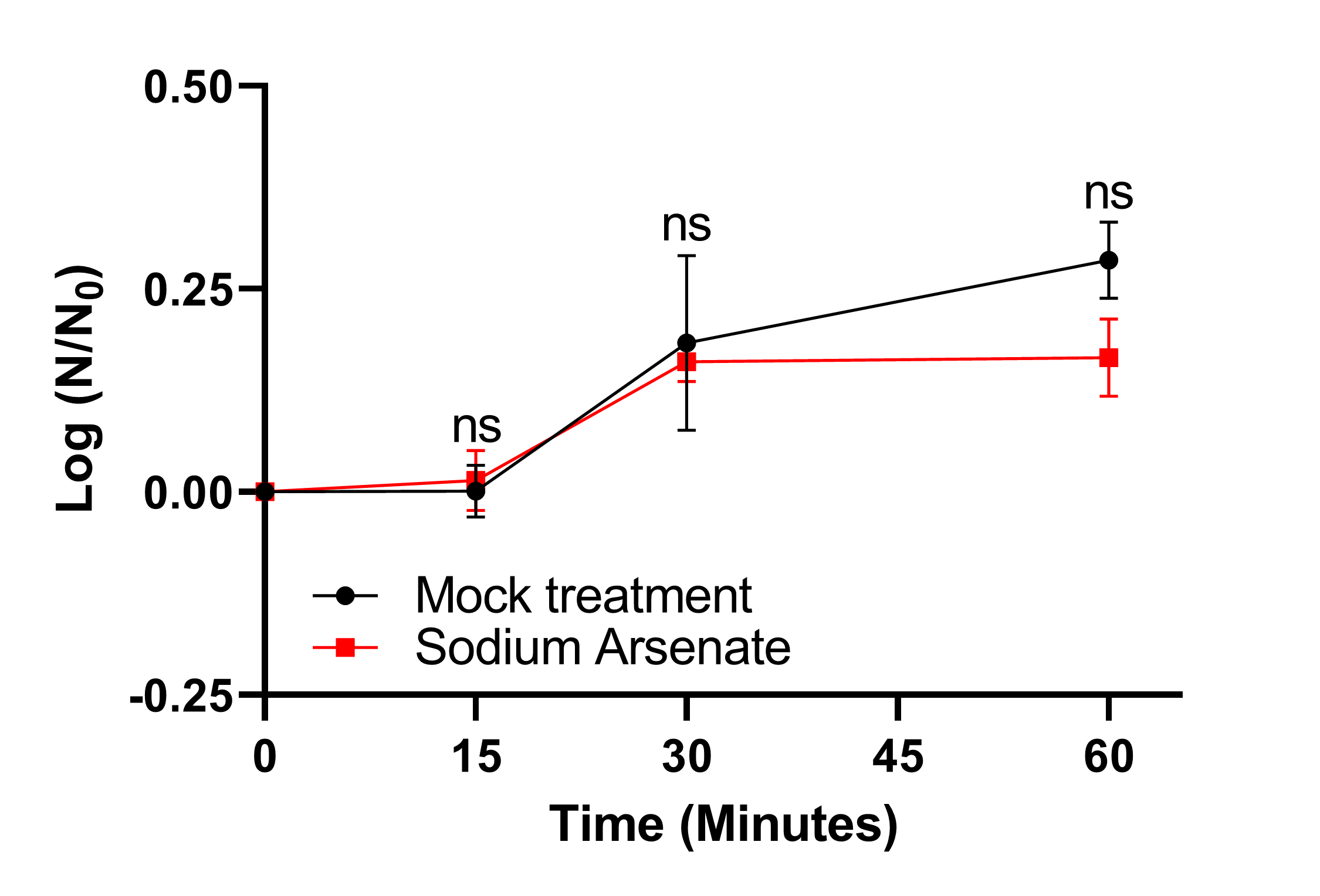

Supplement: S3 Fig — E. faecalis OG1RF cells were grown in BHI until OD600 reached ~0.250. The cultures were split and treated with 10mM arsenate or mock-treated with water for 30 minutes. Following treatment, the cells were washed twice to remove arsenate and resuspended in BHI prior to enumerating colony forming units. Represented are the log ratio of colony forming units ±standard deviations over time. ns = not significant. n = 3. (TIF) [file pone.0254796.s003.tif]

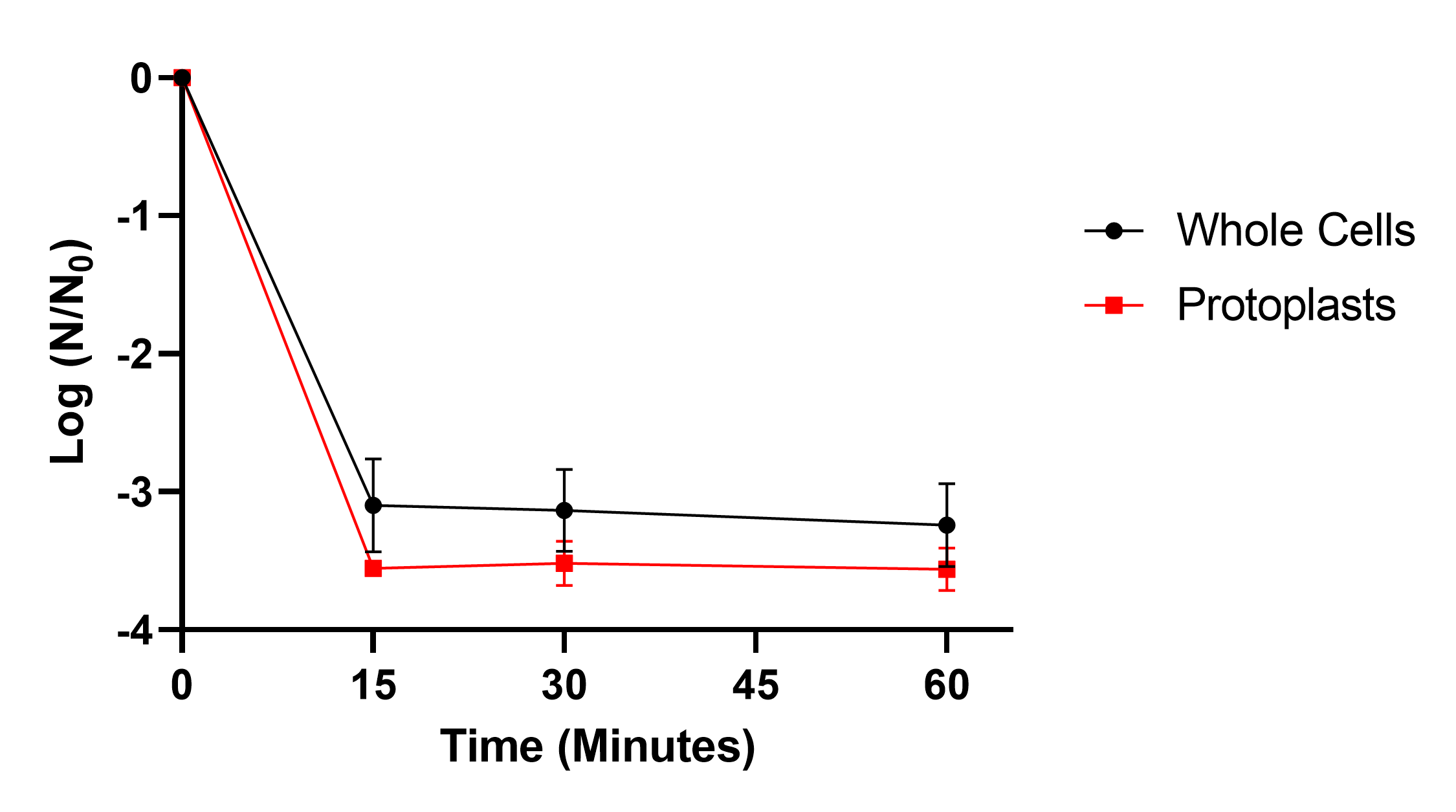

Supplement: S4 Fig — E. faecalis OG1RF cells were grown to mid-logarithmic phase (OD600 ~ 0.3–0.4), split and treated with either lysozyme to generate protoplasts or solvent control to leave cell whole. Subsequently washed cells were subjected to killing by 0.05% SDS. Shown are the average log ratios of survivors ± standard deviations for n = 3 replicates. No significant differences were observed. Exact P-values are reported in S4 Table. (TIF) [file pone.0254796.s004.tif]

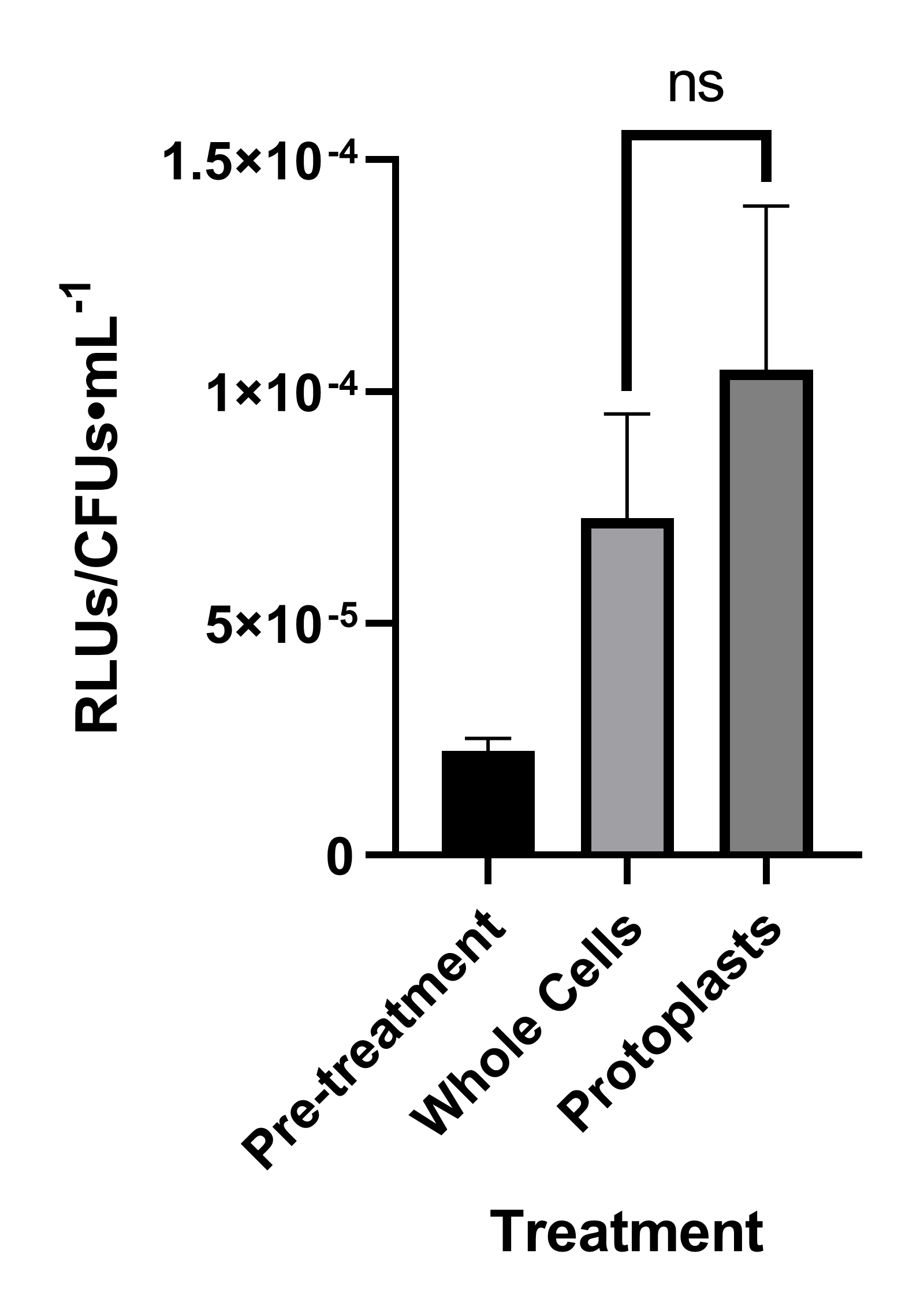

Supplement: S5 Fig — Cells were grown to mid-logarithmic phase (OD600 ~ 0.3–0.4) and treated with either lysozyme to generate protoplasts or solvent control to leave cells whole. ATP levels of pre-treatment cells and whole cells or protoplasts were measured using the Promega BacTiterGlo kit and normalized to colony forming units. Shown are the average relative light units (RLUs) normalized to colony forming units ± standard deviations for n = 3 replicates. ** P = 0.01–0.001; exact P-values are reported in S4 Table. (TIF) [file pone.0254796.s005.tif]

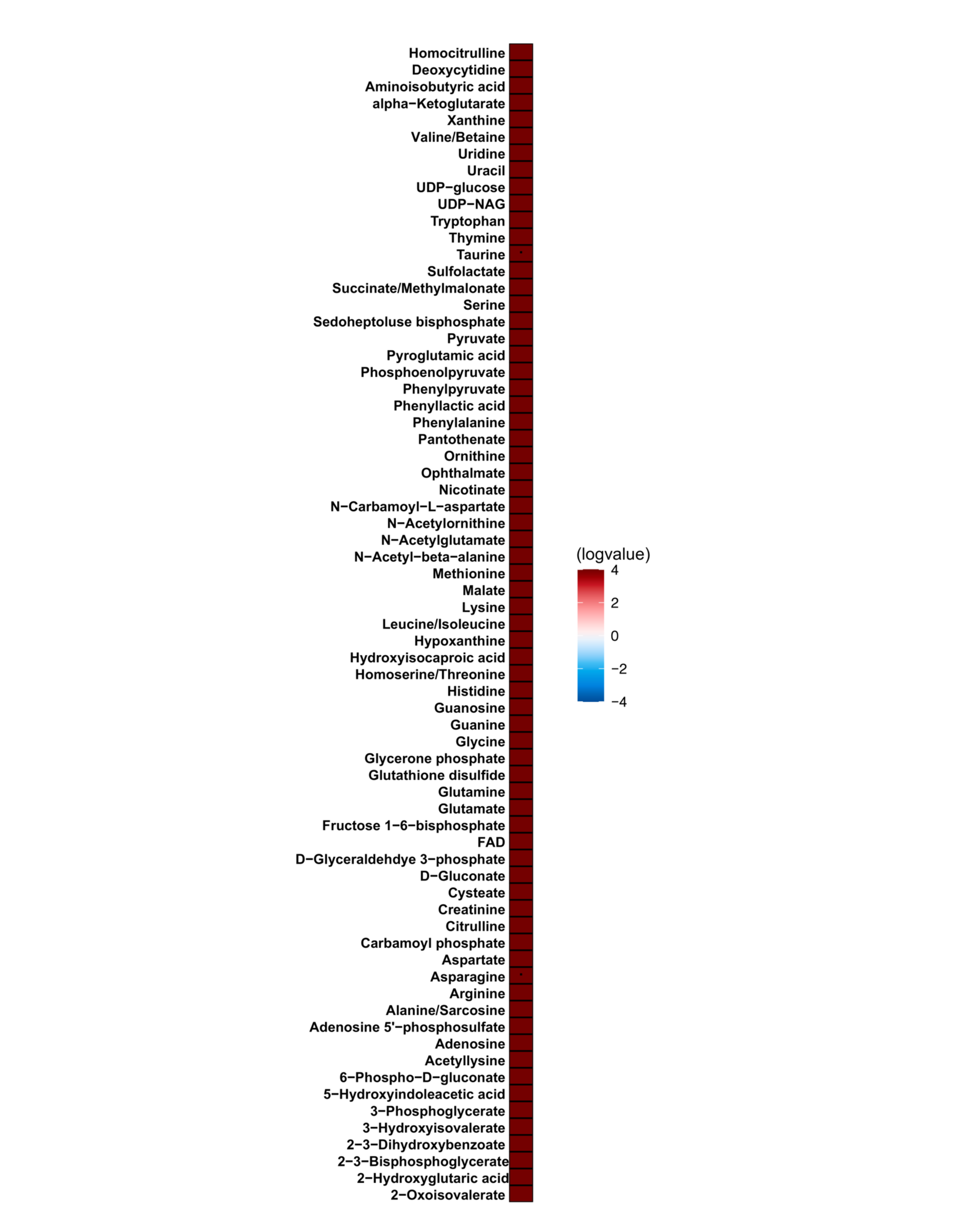

Supplement: S6 Fig — Exponentially growing (OD600 ~ 0.3) E. faecalis cells were grown to mid-logarithmic phase (OD600 ~ 0.3–0.4), split and treated with either lysozyme to generate protoplasts or solvent control to leave cell whole as described in Materials and methods. Metabolites of the cells were extracted and detected via mass spectrometry as outlined in Materials and methods. Represented are the log-transformed fold changes of normalized whole cell metabolites to normalized protoplast metabolites. Normalization was performed dividing raw metabolite data by the ratio of colony forming units of protoplast versus whole cells. n = 5 replicates. (TIF) [file pone.0254796.s006.tif]
