# Supplementary material for: Removal of peptidoglycan and inhibition of active cellular processes leads to daptomycin tolerance in Enterococcus faecalis
Source: PLoS One. 2021 Jul 23;16(7):e0254796. doi: 10.1371/journal.pone.0254796 (PMC8301656; doi:10.1371/journal.pone.0254796)
Supplement: S1 Table — (DOCX) [file pone.0254796.s007.docx]

**S1 Table. List of bacterial strains used in this study.**

| **Bacterial strain** | **Reference** |
| --- | --- |
| *Enterococcus faecalis* OG1RF | Lab stock. |
| *Enterococcus faecium* DO (TX0016) | Generous gift of Barbara Murray[1]. |
| *Staphylococcus aureus* USA300 | From ATCC[2]. |
| *Bacillus subtilis sub. subtilis* 168 | Lab stock. |

**Supplementary Bibliography**

1. Arduino RC, Jacques-Palaz K, Murray BE, Rakita RM. Resistance of *Enterococcus faecium* to neutrophil-mediated phagocytosis. Infect Immun. 1994;62(12):5587-94. Epub 1994/12/01. doi: 10.1128/iai.62.12.5587-5594.1994. PubMed PMID: 7960141; PubMed Central PMCID: PMCPMC303306.

2. Diep BA, Gill SR, Chang RF, Phan TH, Chen JH, Davidson MG, et al. Complete genome sequence of USA300, an epidemic clone of community-acquired meticillin-resistant *Staphylococcus aureus*. Lancet. 2006;367(9512):731-9. Epub 2006/03/07. doi: 10.1016/s0140-6736(06)68231-7. PubMed PMID: 16517273.
