# Supplementary material for: Removal of peptidoglycan and inhibition of active cellular processes leads to daptomycin tolerance in Enterococcus faecalis
Source: PLoS One. 2021 Jul 23;16(7):e0254796. doi: 10.1371/journal.pone.0254796 (PMC8301656; doi:10.1371/journal.pone.0254796)
Supplement: S2 Table — Minimal inhibitory concentrations (MICs) were performed using broth dilution technique. The ranges observed are reported above. Brain heart infusion + 1.5 mM CaCl2 was used for Enterococcus and Staphylococcus strains grown statically. LB + 1.5 mM CaCl2 was used for B. subtilis grown shaking (250 RPM). n = 3 for all experiments. (DOCX) [file pone.0254796.s008.docx]

**S2 Table. Minimal inhibitory concentrations of daptomycin for the bacterium used in this study.**

| **Bacterial strain** | **MIC range (µg/mL)** |
| --- | --- |
| *Enterococcus faecalis* OG1RF | 2-4 |
| *Enterococcus faecium* DO (TX0016) | 4 |
| *Staphylococcus aureus* USA300 | 0.5-1 |
| *Bacillus subtilis sub. subtilis* 168 | 0.5 |

Minimal inhibitory concentrations (MICs) were performed using broth dilution technique. The ranges observed are reported above. Brain heart infusion + 1.5 mM CaCl_2_ was used for *Enterococcus* and *Staphylococcus* strains grown statically. LB + 1.5 mM CaCl_2_ was used for *B. subtilis* grown shaking (250 RPM). *n* = 3 for all experiments.
