# Supplementary material for: Removal of peptidoglycan and inhibition of active cellular processes leads to daptomycin tolerance in Enterococcus faecalis
Source: PLoS One. 2021 Jul 23;16(7):e0254796. doi: 10.1371/journal.pone.0254796 (PMC8301656; doi:10.1371/journal.pone.0254796)
Supplement: S3 Table — (DOCX) [file pone.0254796.s009.docx]

**S3 Table. Colony forming units per milliliter (CFU/mL) taken during cell survival assays within this study.**

| **Variable** | **Corresponding figure in text** | **Time** | **CFU/mL (replicate 1)** | **CFU/mL (replicate 2)** | **CFU/mL (replicate 3)** |
| --- | --- | --- | --- | --- | --- |
| Exponential | Figure 1A | 0 minutes | 2.54 X 10^8^ | 2.71 X 10^8^ | 2.92 X 10^8^ |
|  |  | 15 minutes | 6.50 X 10^4^ | 1.40 X 10^5^ | 3.00 X 10^4^ |
|  |  | 30 minutes | 1.81 X 10^4^ | 1.71 X 10^4^ | 8.40 X 10^3^ |
|  |  | 60 minutes | 4.80 X 10^3^ | 4.10 X 10^3^ | 4.60 X 10^3^ |
|  |  | 240 minutes | 1.00 X 10^2^ | 2.30 X 10^2^ | 2.30 X 10^2^ |
|  |  | 24 hours | Below limit of detection | Below limit of detection | Below limit of detection |
| Stationary | Figure 1A | 0 minutes | 1.27 X 10^9^ | 1.49 X 10^9^ | 1.42 X 10^9^ |
|  |  | 15 minutes | 8.70 X 10^8^ | 7.00 X 10^8^ | 7.20 X 10^8^ |
|  |  | 30 minutes | 8.70 X 10^8^ | 7.80 X 10^8^ | 5.70 X 10^8^ |
|  |  | 60 minutes | 6.20 X 10^8^ | 5.50 X 10^8^ | 7.30 X 10^8^ |
|  |  | 240 minutes | 4.70 X 10^8^ | 3.00 X 10^8^ | 3.00 X 10^8^ |
|  |  | 24 hours | 6.40 X 10^7^ | 5.00 X 10^7^ | 4.00 X 10^7^ |
| Water | Figure 2A | 0 minutes | 3.50 X 10^8^ | 5.00 X 10^8^ | 3.80 X 10^8^ |
|  |  | 15 minutes | 3.20 X 10^4^ | 5.20 X 10^4^ | 9.20 X 10^3^ |
|  |  | 30 minutes | 1.07 X 10^4^ | 7.80 X 10^3^ | 1.78 X 10^3^ |
|  |  | 60 minutes | 5.90 X 10^3^ | 2.90 X 10^3^ | 9.60 X 10^2^ |
| Arsenate | Figure 2A | 0 minutes | 1.78 X 10^8^ | 1.81 X 10^8^ | 2.08 X 10^8^ |
|  |  | 15 minutes | 9.90 X 10^3^ | 1.13 X 10^4^ | 3.60 X 10^3^ |
|  |  | 30 minutes | 2.21 X 10^3^ | 1.83 X 10^3^ | 7.20 X 10^2^ |
|  |  | 60 minutes | 8.30 X 10^2^ | 9.40 X 10^2^ | 2.70 X 10^2^ |
| Solvent control | Figure 3A | 0 minutes | 2.12 X 10^8^ | 2.97 X 10^8^ | 2.40 X 10^8^ |
|  |  | 15 minutes | 1.61 X 10^3^ | 2.70 X 10^3^ | 2.01 X 10^3^ |
|  |  | 30 minutes | 8.90 X 10^2^ | 8.80 X 10^2^ | 6.50 X 10^2^ |
|  |  | 60 minutes | 4.00 X 10^2^ | 3.10 X 10^2^ | 2.40 X 10^2^ |
| Chloramphenicol | Figure 3A | 0 minutes | 1.54 X 10^8^ | 8.90 X 10^7^ | 8.90 X 10^7^ |
|  |  | 15 minutes | 6.90 X 10^4^ | 1.05 X 10^5^ | 2.80 X 10^5^ |
|  |  | 30 minutes | 4.80 X 10^3^ | 5.50 X 10^3^ | 4.20 X 10^3^ |
|  |  | 60 minutes | 6.60 X 10^2^ | 1.21 X 10^3^ | 1.96 X 10^3^ |
| Solvent control | Figure 4A | 0 minutes | 1.15 X 10^8^ | 1.26 X 10^8^ | 1.52 X 10^8^ |
|  |  | 15 minutes | 7.30 X 10^3^ | 5.90 X 10^3^ | 1.09 X 10^3^ |
|  |  | 30 minutes | 4.70 X 10^2^ | 5.20 X 10^2^ | 3.30 X 10^2^ |
|  |  | 60 minutes | 9.00 X 10^1^ | 1.10 X 10^2^ | 1.90 X 10^2^ |
| Cerulenin | Figure 4A | 0 minutes | 8.50 X 10^7^ | 7.90 X 10^7^ | 1.02 X 10^8^ |
|  |  | 15 minutes | 6.30 X 10^4^ | 4.60 X 10^4^ | 5.10 X 10^4^ |
|  |  | 30 minutes | 5.20 X 10^3^ | 2.80 X 10^3^ | 4.70 X 10^3^ |
|  |  | 60 minutes | 6.10 X 10^2^ | 1.80 X 10^2^ | 1.61 X 10^3^ |
| Whole cells | Figure 5A | 0 minutes | 3.15 X 10^8^ | 3.25 X 10^8^ | 2.53 X 10^8^ |
|  |  | 15 minutes | 4.10 X 10^3^ | 2.60 X 10^3^ | 1.72 X 10^4^ |
|  |  | 30 minutes | 1.00 X 10^3^ | 2.40 X 10^2^ | 2.65 X 10^3^ |
|  |  | 60 minutes | 1.20 X 10^2^ | 1.20 X 10^2^ | 1.50 X 10^2^ |
| Protoplasts | Figure 5A | 0 minutes | 3.20 X 10^6^ | 9.30 X 10^5^ | 4.40 X 10^6^ |
|  |  | 15 minutes | 9.00 X 10^5^ | 9.10 X 10^4^ | 7.00 X 10^5^ |
|  |  | 30 minutes | 6.90 X 10^5^ | 4.20 X 10^4^ | 3.70 X 10^5^ |
|  |  | 60 minutes | 3.40 X 10^5^ | 3.09 X 10^4^ | 2.09 X 10^5^ |
| Whole Cells (Exp) | Figure 5B | 0 minutes | 4.50 X 10^8^ | 4.70 X 10^8^ | 3.90 X 10^8^ |
|  |  | 15 minutes | 4.40 X 10^2^ | 2.90 X 10^2^ | 3.40 X 10^2^ |
|  |  | 30 minutes | 3.20 X 10^2^ | 3.20 X 10^2^ | 3.50 X 10^2^ |
|  |  | 60 minutes | 3.30 X 10^2^ | 3.10 X 10^2^ | 3.10 X 10^2^ |
| Protoplasts (Exp) | Figure 5B | 0 minutes | 9.60 X 10^5^ | 3.40 X 10^6^ | 9.00 X 10^5^ |
|  |  | 15 minutes | 3.20 X 10^5^ | 1.12 X 10^6^ | 2.60 X 10^5^ |
|  |  | 30 minutes | 3.90 X 10^5^ | 7.10 X 10^5^ | 2.70 X 10^5^ |
|  |  | 60 minutes | 2.45 X 10^5^ | 4.30 X 10^5^ | 1.82 X 10^5^ |
| Whole Cells (Stat) | Figure 5B | 0 minutes | 9.20 X 10^8^ | 1.25 X 10^9^ | 1.25 X 10^9^ |
|  |  | 15 minutes | 2.56 X 10^8^ | 3.60 X 10^8^ | 4.20 X 10^8^ |
|  |  | 30 minutes | 1.81 X 10^8^ | 1.79 X 10^8^ | 2.53 X 10^8^ |
|  |  | 60 minutes | 3.20 X 10^7^ | 1.63 X 10^8^ | 2.01 X 10^8^ |
| Protoplasts (Stat) | Figure 5B | 0 minutes | 2.80 X 10^8^ | 5.40 X 10^8^ | 2.50 X 10^8^ |
|  |  | 15 minutes | 3.00 X 10^8^ | 3.15 X 10^8^ | 3.00 X 10^8^ |
|  |  | 30 minutes | 3.50 X 10^8^ | 3.14 X 10^8^ | 2.75 X 10^8^ |
|  |  | 60 minutes | 2.11 X 10^8^ | 2.40 X 10^8^ | 2.15 X 10^8^ |
| Whole Cells  [*E. faecium*] | Figure 6A | 0 minutes | 1.21 X 10^8^ | 2.40 X 10^8^ | 3.10 X 10^7^ |
|  |  | 15 minutes | 6.30 X 10^3^ | 5.40 X 10^3^ | 6.40 X 10^3^ |
|  |  | 30 minutes | 2.81 X 10^3^ | 2.25 X 10^3^ | 2.71 X 10^3^ |
|  |  | 60 minutes | 2.00 X 10^2^ | 6.40 X 10^2^ | 7.20 X 10^2^ |
| Protoplasts [*E. faecium*] | Figure 6A | 0 minutes | 4.80 X 10^6^ | 9.70 X 10^5^ | 6.90 X 10^5^ |
|  |  | 15 minutes | 2.08 X 10^6^ | 6.00 X 10^5^ | 4.50 X 10^5^ |
|  |  | 30 minutes | 5.30 X 10^5^ | 5.30 X 10^5^ | 3.70 X 10^5^ |
|  |  | 60 minutes | 1.70 X 10^5^ | 5.40 X 10^5^ | 3.70 X 10^5^ |
| Whole Cells  [*B. subtilis*] | Figure 6B | 0 minutes | 1.82 X 10^7^ | 1.76 X 10^7^ | 3.20 X 10^7^ |
|  |  | 15 minutes | 2.10 X 10^2^ | 1.50 X 10^2^ | 1.30 X 10^2^ |
|  |  | 30 minutes | 2.20 X 10^2^ | 1.90 X 10^2^ | 1.30 X 10^2^ |
|  |  | 60 minutes | 2.30 X 10^2^ | 1.20 X 10^2^ | 1.40 X 10^2^ |
| Protoplasts [*B. subtilis*] | Figure 6B | 0 minutes | 2.90 X 10^5^ | 7.00 X 10^5^ | 5.40 X 10^5^ |
|  |  | 15 minutes | 6.50 X 10^4^ | 9.30 X 10^4^ | 1.64 X 10^5^ |
|  |  | 30 minutes | 3.10 X 10^4^ | 4.90 X 10^4^ | 1.32 X 10^5^ |
|  |  | 60 minutes | 4.60 X 10^4^ | 9.40 X 10^4^ | 2.30 X 10^5^ |
| Whole Cells  [*S. aureus*] | Figure 6C | 0 minutes | 4.70 X 10^6^ | 2.81 X 10^7^ | 8.10 X 10^6^ |
|  |  | 15 minutes | 2.74 X 10^3^ | 1.64 X 10^4^ | 2.52 X 10^3^ |
|  |  | 30 minutes | 1.17 X 10^3^ | 2.36 X 10^3^ | 1.70 X 10^3^ |
|  |  | 60 minutes | 2.30 X 10^2^ | 5.10 X 10^2^ | 1.90 X 10^2^ |
| Protoplasts  [*S. aureus*] | Figure 6C | 0 minutes | 1.39 X 10^7^ | 2.28 X 10^7^ | 1.50 X 10^7^ |
|  |  | 15 minutes | 1.22 X 10^7^ | 1.45 X 10^7^ | 1.36 X 10^7^ |
|  |  | 30 minutes | 1.52 X 10^7^ | 1.04 X 10^7^ | 1.12 X 10^7^ |
|  |  | 60 minutes | 1.17 X 10^6^ | 9.10 X 10^5^ | 9.80 X 10^5^ |
| Mock Treatment | S3 Figure | 0 minutes | 3.08 X 10^8^ | 3.40 X 10^8^ | 3.06 X 10^8^ |
|  |  | 15 minutes | 2.90 X 10^8^ | 3.70 X 10^8^ | 3.00 X 10^8^ |
|  |  | 30 minutes | 5.30 X 10^8^ | 3.90 X 10^8^ | 5.50 X 10^8^ |
|  |  | 60 minutes | 6.20 X 10^8^ | 5.80 X 10^8^ | 6.40 X 10^8^ |
| Sodium Arsenate | S3 Figure | 0 minutes | 2.06 X 10^8^ | 2.20 X 10^8^ | 1.95 X 10^8^ |
|  |  | 15 minutes | 2.17 X 10^8^ | 2.07 X 10^8^ | 2.17 X 10^8^ |
|  |  | 30 minutes | 3.00 X 10^8^ | 3.00 X 10^8^ | 2.97 X 10^8^ |
|  |  | 60 minutes | 2.98 X 10^8^ | 2.90 X 10^8^ | 3.20 X 10^8^ |
| Whole Cells | S4 Figure | 0 minutes | 3.13 X 10^8^ | 2.76 X 10^8^ | 3.30 X 10^8^ |
|  |  | 15 minutes | 3.61 X 10^5^ | 3.70 X 10^5^ | 1.07 X 10^5^ |
|  |  | 30 minutes | 3.26 X 10^5^ | 3.13 X 10^5^ | 1.09 X 10^5^ |
|  |  | 60 minutes | 2.72 X 10^5^ | 2.32 X 10^5^ | 8.50 X 10^4^ |
| Protoplasts | S4 Figure | 0 minutes | 7.30 X 10^6^ | 1.01 X 10^6^ | 1.31 X 10^6^ |
|  |  | 15 minutes | 2.07 X 10^3^ | 2.80 X 10^2^ | 3.60 X 10^2^ |
|  |  | 30 minutes | 1.75 X 10^3^ | 4.70 X 10^2^ | 3.30 X 10^2^ |
|  |  | 60 minutes | 1.35 X 10^3^ | 3.70 X 10^2^ | 4.00 X 10^2^ |
