# Supplementary material for: Removal of peptidoglycan and inhibition of active cellular processes leads to daptomycin tolerance in Enterococcus faecalis
Source: PLoS One. 2021 Jul 23;16(7):e0254796. doi: 10.1371/journal.pone.0254796 (PMC8301656; doi:10.1371/journal.pone.0254796)
Supplement: S4 Table — Two-tailed Welch’s t-tests were used to generate P-values. In the text, α = 0.05 was used to determine significance. (DOCX) [file pone.0254796.s010.docx]

**S4 Table. *P-*values determined within this study.**

| **Cell survival assays** | | | | |
| --- | --- | --- | --- | --- |
| **Variable 1** | **Variable 2** | **Corresponding figure in text** | **Time** | **P-value** |
| Exponential | Stationary | Figure 1A | 15 minutes | <0.001 |
|  |  |  | 30 minutes | <0.001 |
|  |  |  | 60 minutes | <0.001 |
| Mock treatment | Sodium arsenate | Figure 2A | 15 minutes | 0.769 |
|  |  |  | 30 minutes | 0.769 |
|  |  |  | 60 minutes | 0.769 |
| Solvent control | Chloramphenicol | Figure 3A | 15 minutes | 0.002 |
|  |  |  | 30 minutes | 0.001 |
|  |  |  | 60 minutes | 0.015 |
| Solvent control | Cerulenin | Figure 4A | 15 minutes | 0.022 |
|  |  |  | 30 minutes | 0.002 |
|  |  |  | 60 minutes | 0.030 |
| Whole Cells | Protoplasts | Figure 5A | 15 minutes | <0.001 |
|  |  |  | 30 minutes | <0.001 |
|  |  |  | 60 minutes | <0.001 |
| Whole Cells (Stat) | Protoplasts (Stat) | Figure 5B | 15 minutes | 0.023 |
|  |  |  | 30 minutes | 0.023 |
|  |  |  | 60 minutes | 0.023 |
| Whole Cells  [*E. faecium*] | Protoplasts  [*E. faecium*] | Figure 6A | 15 minutes | <0.001 |
|  |  |  | 30 minutes | <0.001 |
|  |  |  | 60 minutes | <0.001 |
| Whole Cells  [*B. subtilis*] | Protoplasts  [*B. subtilis*] | Figure 6B | 15 minutes | <0.001 |
|  |  |  | 30 minutes | <0.001 |
|  |  |  | 60 minutes | <0.001 |
| Whole Cells  [*S. aureus*] | Protoplasts  [*S. aureus*] | Figure 6C | 15 minutes | <0.001 |
|  |  |  | 30 minutes | <0.001 |
|  |  |  | 60 minutes | <0.001 |
| Mock Treatment | Sodium Arsenate | S3 Figure | 15 minutes | 0.884 |
|  |  |  | 30 minutes | 0.884 |
|  |  |  | 60 minutes | 0.104 |
| Whole Cells | Protoplasts | S4 Figure | 15 minutes | 0.222 |
|  |  |  | 30 minutes | 0.233 |
|  |  |  | 60 minutes | 0.233 |
| **ATP Determination Assays** | | | | |
| **Variable 1** | **Variable 2** | **Corresponding figure in text** | **P-value** |  |
| Exponential | Stationary | Figure 1B | 0.0246 |  |
| Mock treatment | Arsenate | Figure 2B | 0.0011 |  |
| Solvent control | Chloramphenicol | Figure 3B | 0.5948 |  |
| Solvent control | Cerulenin | Figure 4B | <0.001 |  |
| Whole Cells | Protoplasts | S5 Figure | 0.2675 |  |

Two-tailed Welch’s *t-*tests were used to generate *P*-values. In the text, α = 0.05 was used to determine significance.
